# Exosome-delivered microRNAs modulate the inflammatory response to endotoxin

**DOI:** 10.1038/ncomms8321

**Published:** 2015-06-18

**Authors:** Margaret Alexander, Ruozhen Hu, Marah C. Runtsch, Dominique A. Kagele, Timothy L. Mosbruger, Tanya Tolmachova, Miguel C. Seabra, June L. Round, Diane M. Ward, Ryan M. O'Connell

**Affiliations:** 1 Division of Microbiology and Immunology, Department of Pathology, University of Utah, 4280 JMRB, 15 North Medical Drive East, Salt Lake City, Utah 84112, USA; 2 Huntsman Cancer Institute, University of Utah School of Medicine, Salt Lake City, Utah 84112, USA; 3 Molecular Medicine Section, National Heart and Lung Institute, Imperial College London, London SW7 2AZ, UK

## Abstract

MicroRNAs regulate gene expression posttranscriptionally and function within the cells in which they are transcribed. However, recent evidence suggests that microRNAs can be transferred between cells and mediate target gene repression. We find that endogenous miR-155 and miR-146a, two critical microRNAs that regulate inflammation, are released from dendritic cells within exosomes and are subsequently taken up by recipient dendritic cells. Following uptake, exogenous microRNAs mediate target gene repression and can reprogramme the cellular response to endotoxin, where exosome-delivered miR-155 enhances while miR-146a reduces inflammatory gene expression. We also find that miR-155 and miR-146a are present in exosomes and pass between immune cells *in vivo*, as well as demonstrate that exosomal miR-146a inhibits while miR-155 promotes endotoxin-induced inflammation in mice. Together, our findings provide strong evidence that endogenous microRNAs undergo a functional transfer between immune cells and constitute a mechanism of regulating the inflammatory response.

## Abstract (web summary)

Response to inflammatory stimuli such as endotoxin is coordinated at many levels. Here, the authors show that two microRNAs known to regulate inflammatory response inside the cell are secreted by dendritic cells and modulate inflammatory signalling in the neighbouring cells.

## Graphical abstract

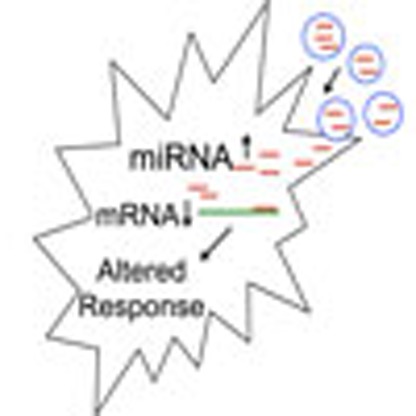

Intercellular communication is essential for immune cells to coordinate inflammatory responses. Cytokines, chemokines and cell surface receptors are well-known mediators of this process. In addition to these classical signalling molecules, emerging evidence suggests that immune cells can signal by secreting small lipid packages called exosomes, which carry a variety of different molecules that can be taken up by recipient cells^1^,^2^,^3^,^4^. The functional relevance of exosomes in many different biological systems, including the immune system, is beginning to be demonstrated^5^,^6^,^7^,^8^,^9^,^10^,^11^.

MicroRNAs (miRNAs) are important modulators of gene expression that function by targeting messenger RNAs for degradation or preventing translation. Typically, miRNAs are thought to function within the cells in which they are made; however, recently, miRNAs have been observed in secreted exosomes^12^,^13^,^14^. Immune cells, including antigen-presenting dendritic cells (DCs) and T lymphocytes, can both secrete and take up exosomal miRNAs, suggesting that exosomal transfer of miRNAs could be a novel mechanism for intercellular communication^12^,^14^,^15^. Furthermore, recent studies indicate that the loading of miRNAs into exosomes is a selective process where specific motifs in miRNA sequences are recognized by the RNA-binding protein, hnRNPA2B1 (ref. 16). Other reports find that miRNA loading into exosomes is dependent on 3′-end uridylated isoforms^17^ and on the levels of miRNA targets in producer cells^18^. Consistent with this, exosomal miRNA signatures do not simply reflect the miRNA composition of the parent cell, but are composed of a distinct set of miRNAs^16^,^18^,^19^,^20^,^21^. This argues that certain miRNAs have evolved to be packaged into exosomes to carry out their biological functions.

Exosomally transferred miRNAs are emerging as novel regulators of cellular function. There is evidence in both immune cells and other cell types that transferred miRNAs repress target mRNAs in recipient cells^12^,^13^,^14^,^22^,^23^,^24^. The transfer of miRNAs can also cause physiological changes in recipient cells^5^,^6^,^7^, as demonstrated by miRNAs moving from cancer cells to endothelial cells, which promotes tumour metastasis^5^. Cancer cells can also receive exosomal miRNAs secreted from immune cells, which were shown to have an anti-proliferative effect on the tumour cells^7^. These data suggest that different cell types can secrete or receive miRNAs as a form of communication and have set the stage for investigating the functional roles of transferred miRNAs in the context of immune responses.

Within the immune system, several specific miRNAs have recently emerged as important regulators of immune cell function. Among these, miR-155 is a promoter of inflammatory responses, while miR-146a is a mediator of immune suppression^25^,^26^,^27^,^28^. Despite significant progress in our understanding of how these miRNAs influence immunity *in vivo*, there are many aspects of their regulation and function that remain unclear. In the current study, we investigate whether endogenous miR-155 and miR-146a are functionally transferred between primary bone marrow-derived DCs (BMDCs). We find that both of these miRNAs are released within exosomes and are taken up by recipient BMDCs. On uptake, the miRNAs are associated with Ago proteins, knock down their respective targets and reprogramme the response of BMDCs to endotoxin challenge. We also show that miR-155 can be transferred between immune cells *in vivo*. Finally, we demonstrate that injection of miR-146a-containing exosomes into mice inhibits their inflammatory response to endotoxin, whereas injection of miR-155-containing exosomes promotes inflammation following exposure to the same inflammatory stimulus. Our study supports a model whereby exosomal miRNAs participate in the regulation of inflammatory responses.

## Results

### miR-155 is found in exosomes and transferred between BMDCs

**Figure 1:**
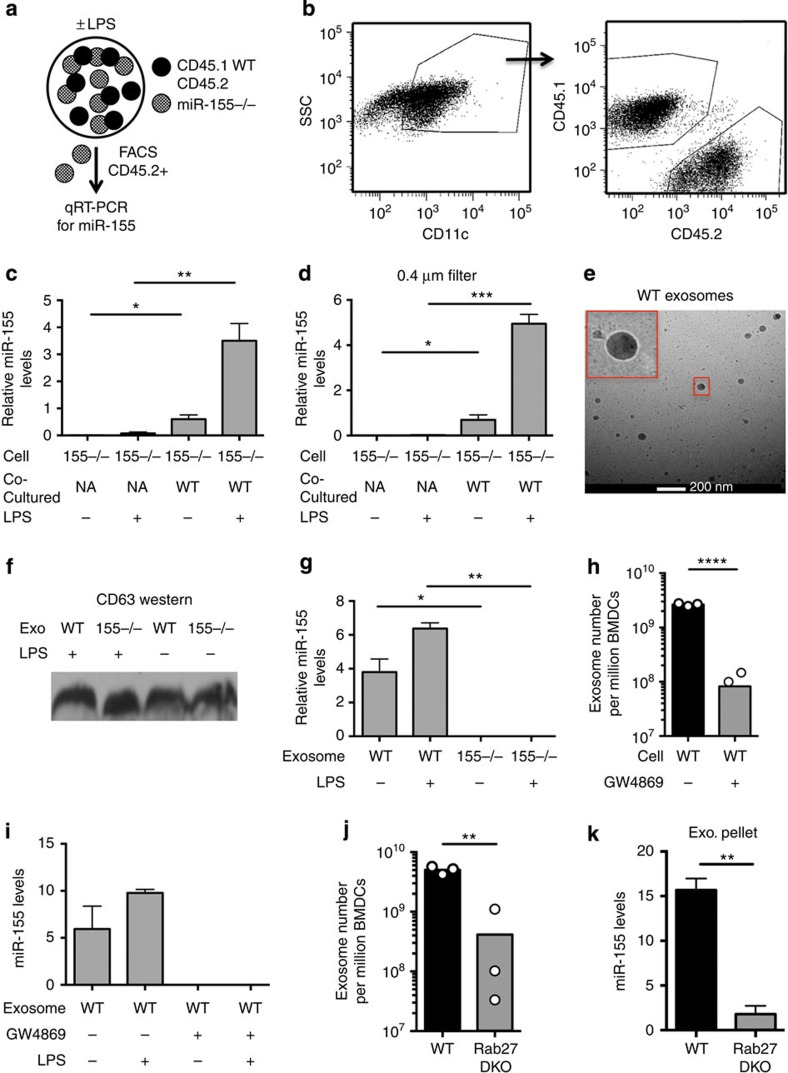
miR-155 is transferred between BMDCs and is present in exosomes. (**a**) A schematic of the co-culture experiment. (**b**) Representative FACS plots where co-cultured *CD45.1*^+^ *Wt* and *CD45.2*^*+*^ *miR-155−/−* CD11c^+^ BMDCs were separated (*n*=4). (**c**) Relative miR-155 levels were quantified via qRT–PCR from isolated *miR-155−/−* BMDCs that had been cultured alone or with *Wt* BMDCs in the presence or the absence of LPS for 24 h (*n*=4). (**d**) Relative miR-155 levels were measured via qRT–PCR in *miR-155−/−* BMDCs either cultured alone or with *Wt* BMDCs separated by a 0.4-μm filter for 24 h with or without LPS (*n*=3). (**e**) Cryo-EM of exosomes isolated from *Wt* BMDCs. Scale bar, 200 nm. Red box is enlarged in the upper left corner. (**f**) CD63 protein levels in the exosomal pellet from *Wt* and *miR-155−/−* BMDCs treated with or without LPS. (**g**) Relative levels of miR-155 in exosomes derived from *Wt* or *miR-155−/−* BMDCs treated with or without LPS (*n*=3). (**h**) Exosome quantification of *Wt* BMDCs treated with or without GW4869 (*n*=3). Limit of detection is 2 × 10^7^ exosomes. (**i**) Relative levels of miR-155 were measured in the exosomal pellet from *Wt* BMDCs treated with or without LPS and GW4869 as quantified by qRT–PCR (*n*=2). (**j**) Exosome quantification of *Wt* and *Rab27 DKO* BMDC-derived exosomes (*n*=2). Limit of detection is 2 × 10^7^ exosomes. (**k**) miR-155 levels in exosome pellets from *Wt* and *Rab27 DKO* BMDC-conditioned medium (*n*=2). Data represent two independent experiments and are presented as the mean±s.d. (error bars). *P<0.05; **P<0.01, Student's *t*-test.

miR-155 is an immunomodulatory miRNA expressed by many types of immune cells including DCs^27^. We sought to determine whether miR-155 could be passed between cultured BMDCs. Co-cultures of primary mouse BMDCs derived from *CD45.1*^+^ *Wt* mice and *CD45.2*^*+*^ *miR-155−/−* mice were set up at a 1:1 ratio with and without lipopolysaccharide (LPS) treatment (Fig. 1a). As a control, *miR-155−/−* BMDCs were also cultured under the same conditions without *Wt* cells. After 24 h, the co-cultured *Wt* and *miR-155−/−* CD11c^+^ BMDCs were separated based on their differential CD45 markers using fluorescence-activated cell sorting (FACS) (Fig. 1b). Quantitative reversetranscriptase–PCR (qRT–PCR) was performed on RNA isolated from the CD45.2^+^ *miR-155−/−* BMDCs. miR-155 was detected in *miR-155−/−* BMDCs that were cultured with *Wt* cells and the signal was clearly above background levels established using *miR-155−/−* BMDCs cultured alone (Fig. 1c). When cells were treated with LPS, the transfer of miR-155 to *miR155−/−* cells was increased, consistent with previous findings that cellular miR-155 concentrations are elevated following LPS stimulation^29^ (Fig. 1c).

To determine whether cell–cell contact is necessary for the transfer of miR-155, we used 0.4-μm filters to separate *miR-155−/−* and *Wt* BMDCs that were co-cultured in the presence or the absence of LPS for 24 h. The 0.4-μm pore size allows for small molecules and vesicles such as exosomes to pass through but prevents cell-contact-mediated exchange of material^23^. We detected miR-155 in the *miR-155−/−* BMDCs that were cultured with *Wt* BMDCs, which was above background (Fig. 1d). Our data indicate that miR-155 is passed between cells, and that cell–cell contact is not necessary for transfer to occur between BMDCs.

As miRNAs have recently been shown to be transferred between immune cells within exosomes, we investigated whether miR-155 is contained within these secreted vesicles. To address this question, we isolated the exosomal pellet from *Wt* or *miR-155−/−* BMDC conditioned media using differential centrifugation. Both electron microscopy (EM) and a CD63 western blotting of the isolated vesicles indicated that we had successfully isolated exosomes (Fig. 1e,f). Using qRT–PCR, we found that miR-155 was contained in exosomes derived from *Wt* BMDCs but not in exosomes derived from *miR-155−/−* cells (Fig. 1g). BMDCs treated with LPS enhanced the levels of miR-155 found in the exosomal pellet, consistent with higher levels of miR-155 being produced by the activated BMDCs. In addition, we blocked exosome formation by treating donor BMDCs with GW4869, a drug that hinders exosome biogenesis by blocking neutral sphingomyelinase 2 (nSMase2) (refs 13, 15). Following drug treatment, the pellet contained significantly reduced exosomes as determined by EXOCET quantification (Fig. 1h), CD63 western blotting and EM (Supplementary Fig. 1). Drug treatment also prevented the detection of miR-155 in the exosomal pellet (Fig. 1i), suggesting that miR-155 is contained within exosomes. In addition, we derived BMDCs from *Rab27a* and *Rab27b* double-knockout mice (*Rab27 DKO*), which have been previously shown to have decreased release of exosomes^23^. We found that *Rab27 DKO* BMDCs had both decreased exosome release (Fig. 1j) and a corresponding decrease in miR-155 in the exosomal pellet (Fig. 1k). Together, these data show that miR-155 can be passed between BMDCs, and that miR-155 is contained in exosomes produced by BMDCs.

### Exosomal transfer of miR-155 is functionally relevant

**Figure 2:**
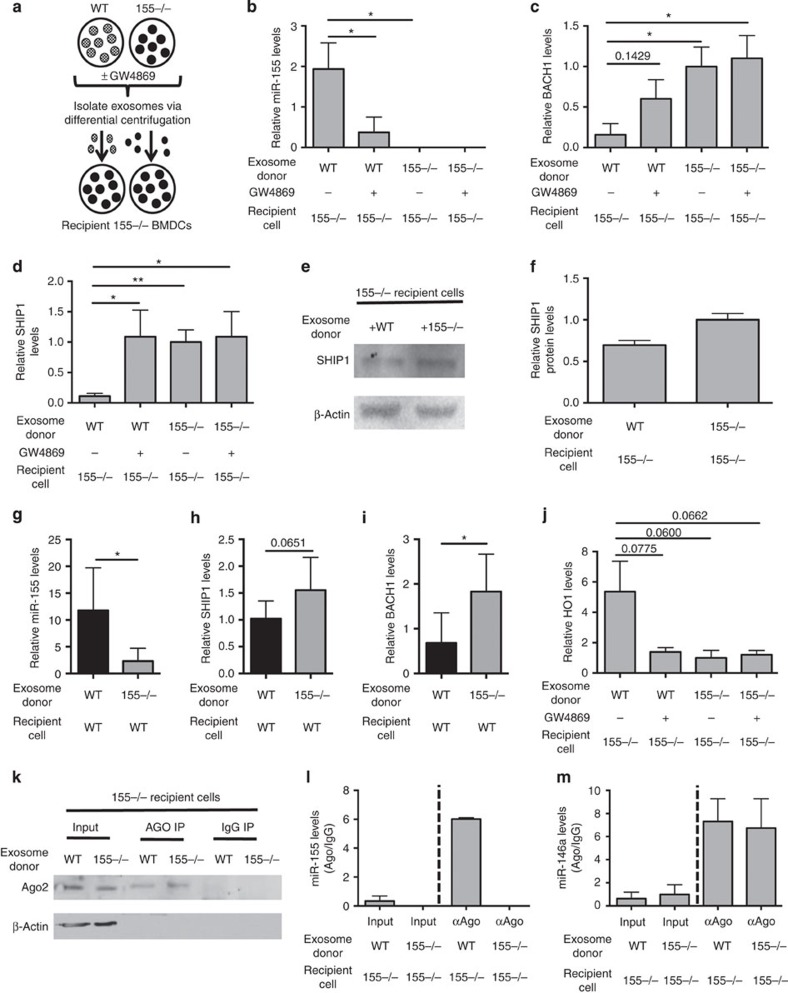
Functional transfer of miR-155 via exosomes *in vitro*. (**a**) Schematic of the exosome transfer experiment. (**b**) qRT–PCR was used to measure relative miR-155 levels in *miR-155−/−* BMDCs that received either *Wt* or *miR-155−/−* exosomes derived from BMDCs treated with or without GW4869 (*n*=5). (**c**,**d**) mRNA levels of miR-155 targets, BACH1 and SHIP1, from the same experiment shown in **b** as measured by qRT–PCR (*n*=5). (**e**) Representative western blottings of SHIP1 and β-actin in *miR-155−/−* BMDCs given either *Wt* or *miR-155−/−* exosomes. (**f**) Protein levels of SHIP1 were quantified using ImageJ software (*n*=2). (**g**) Relative miR-155 levels in *Wt* BMDCs given either *Wt* or *miR-155−/−* exosomes as quantified by qRT–PCR (*n*=6). (**h**,**i**) BACH1 and SHIP1 mRNA levels were measured in the same experiment shown in **g** as quantified by qRT–PCR (*n*=6). (**j**) qRT–PCR was used to quantify HO1 mRNA levels during the experiment in **b** (*n*=5). (**k**) Western blotting for AGO2 and β-actin from *miR-155−/−* BMDCs given *Wt* or *miR-155−/−* exosomes. On the left is the input (whole-cell lysate), the middle is from the pan-AGO pulldown where one-third of input was used and the right is the IgG pulldown where one-third of the input was used. (**l**) Relative miR-155 levels were quantified via qRT–PCR in the same experiment shown in **k**. (**m**) miR-146a levels were quantified using qRT–PCR during the experiment in **k**. Levels in **l**,**m** are plotted as Ago:IgG. Dotted line separates input from pull-down groups. Data represent two independent experiments and are presented as the mean±s.d. (error bars). *P<0.05; **P<0.01, Student's *t*-test.

With the knowledge that miR-155 can be transferred between BMDCs, we wanted to determine whether exosomes are sufficient for this transfer and whether transfer could result in knockdown of target mRNAs. To specifically investigate the impact of exosomally transferred miRNA without the effects of other factors that are released from BMDCs, we purified exosomes away from other components in the conditioned medium using differential centrifugation and washing. Next, the exosomes were re-suspended in fresh medium and administered to recipient cells. *Wt* (1 × 10^6^) or *miR-155−/−* BMDCs produced ∼5 × 10^8^ exosomes in 24 h (Supplementary Fig. 2). Exosomes isolated from the supernatant of both *Wt* and *miR-155−/−* BMDCs treated with GW4869 or dimethylsulfoxide vehicle control were transferred to *miR-155−/−* receipient BMDCs. *miR-155−/−* recipient BMDCs were incubated with donor exosomes for 24 h, to allow time for miRNA transfer and knockdown of miRNA targets (Fig. 2a). Using qRT–PCR, we detected increased miR-155 levels and decreased mRNA levels of miR-155 targets BACH1 and SHIP1 when cells were treated with *Wt* exosomes (Fig. 2b–d). These changes were prevented if the exosomes were derived from *miR-155−/−* BMDCs, or if the *Wt* donor cells were pretreated with GW4869. SHIP1 protein levels were also decreased in *miR-155−/−* BMDCs that received *Wt* exosomes (Fig. 2e,f). Exosome delivery of miR-155 brought its levels in the *miR-155−/−* recipient cells to ∼20% of *Wt* miR-155 levels (Supplementary Fig. 3a,b). Furthermore, we also looked at the relative expression of a separate miRNA, miR-425, which has been previously seen to be released in exosomes^30^, as a control. The levels of miR-425 increased with exosome delivery and were roughly the same in *Wt* and knockout groups (Supplementary Fig. 3c,d).

In addition to using *miR-155−/−* recipient cells, which provide a clean background to clearly detect the transferred miRNA, we also examined whether transferred miR-155 could be detected in *Wt* BMDC recipients. miR-155 levels were increased on treatment of *Wt* BMDCs with *Wt* exosomes but were not increased when treated with *miR-155−/−* exosomes (Fig. 2g). In addition, the mRNA levels of both BACH1 and SHIP1 were decreased in *Wt* BMDCs receiving exosomal miR-155 (Fig. 2h,i). These data indicate that miR-155 can be transferred between *Wt* BMDCs in exosomes, resulting in the knockdown of known miR-155 targets.

As we have found that miR-155 can knock down its targets in recipient cells, we next investigated whether the transfer of miR-155 alters downstream factors in recipient cells. We found that exosomal miR-155 increased the expression of *HO1* (Fig. 2j), an oxidative stress response gene that is well known to be repressed by *BACH1*, a gene we have shown is targeted by transferred miR-155 (ref. 29; Fig. 2c). These results indicate that transferred miR-155 not only represses its putative direct targets but can also affect factors that are downstream of these targets.

To further characterize the functional transfer of miR-155 between BMDCs, we examined whether transferred miR-155 is associated with AGO proteins that are essential for miRNA-mediated knockdown of targets. Following exosomal transfer of miR-155 into *miR-155−/−* BMDCs, an AGO immunoprecipitation (IP) was performed using a pan-AGO antibody and western blotting for AGO2 was preformed to verify pulldown was occurring. Using qRT–PCR, we found that miR-155 is associated with AGO proteins in *miR-155−/−* recipient cells (Fig. 2k,l). We did not detect miR-155-associated AGO proteins when *miR-155−/−* BMDCs were treated with *miR-155−/−* exosomes. Further, AGO2 was not detected via western blotting and miR-155 was not pulled down when an isotype control antibody was used. As an additional control, we found that another miRNA, miR-146a, was also enriched in the AGO pulldown from both groups (Fig. 2m). These data demonstrate that exosomal miR-155 is associated with AGO proteins, key components of the RNA-induced silencing complex (RISC) complex, following its uptake by recipient BMDCs.

### Exosomal transfer of miR-146a is functionally relevant

**Figure 3:**
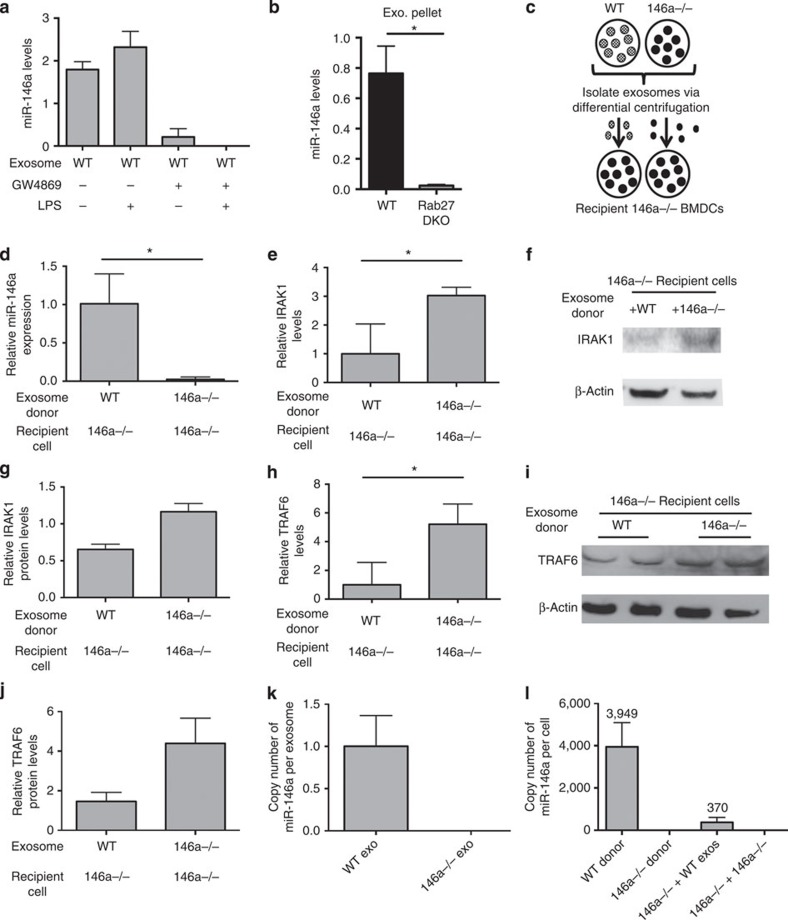
Functional transfer of miR-146a via exosomes *in vitro*. (**a**) Levels of miR-146a in the exosomal pellet derived from BMDCs that were treated with or without GW4869 and LPS (*n*=2). (**b**) miR-146a levels in *Wt* and *Rab27 DKO* BMDC-derived exosomal pellets (*n*=2). (**c**) Schematic of miR-146a exosome-transfer experiment where *Wt* or *miR-146a−/−* exosomes were isolated from BMDCs and transferred to recipient *miR-146a−/−* BMDCs. RNA was isolated after 24 h and the presence of miR-146a was assayed via qRT–PCR. (**d**) Relative levels of miR-146a in *miR-146a−/−* BMDCs given exosomes derived from *Wt* or *miR-146a−/−* BMDCs (*n*=4). (**e**) mRNA levels of miR-146a target, IRAK1, were measured from the same cells as in **d** via qRT–PCR (*n*=4). (**f**) Representative western blottings of IRAK1 and β-actin from *miR-146a−/−* cells given either *Wt* or *miR-146a−/−* exosomes. (**g**) IRAK1 protein levels were quantified using ImageJ software (*n*=2). (**h**) mRNA levels of miR-146a target, TRAF6, were measured in the same cells as in **d** via qRT–PCR. (**i**) Western blottings for TRAF6 and β-actin from *miR-146a−/−* BMDCs given either *Wt* or *miR-146a−/−* exosomes (*n*=2). (**j**) Western blotting results are quantified with ImageJ software. (**k**) Copy number of miR-146a in *Wt* and *miR-146a−/−* exosomes (*n*=3). Copy number is calculated based on a standard curve where a known amount of synthetic miR-146a was spiked into *miR-146a−/−* BMDC-derived exosome pellet followed by RNA isolation and qRT–PCR. (**l**) Copy number of miR-146a was measured via qRT–PCR in *miR-146a−/−* recipient BMDCs that received either *Wt* or *miR-146a−/−* exosomes (146a−/− BMDC+*Wt* exos and 146a−/− BMDC+146a−/− exos), as well as in *Wt* and *miR-146a−/−* donor BMDCs (*n*=3). Average copy number is displayed above. Copy number is calculated based on a standard curve where a known amount of synthetic miR-146a was spiked into *miR-146a−/−* BMDC pellet followed by RNA isolation and qRT–PCR. Data represent two independent experiments and are presented as the mean±s.d. (error bars). *P<0.05, Student's *t*-test.

miR-146a is an important anti-inflammatory miRNA involved in DC function^27^ and plays an opposing role to miR-155 during inflammatory responses^25^,^31^. We wanted to determine whether miR-146a was also contained in BMDC-derived exosomes; hence, we isolated exosomes from *Wt* BMDCs that had been treated with or without GW4869 and/or LPS, and found that miR-146a was contained in exosomes from untreated BMDCs but was not present in the exosomal pellet from BMDCs treated with GW4869 (Fig. 3a). miR-146a is marginally increased in exosomes from BMDCs treated with LPS. In addition, reductions in miR-146a were observed in the extracellular exosomal fraction obtained from *Rab27 DKO* BMDCs compared with *Wt* controls (Fig. 3b). These data reveal that miR-146a is contained within exosomes released from BMDCs.

To test whether miR-146a could be functionally transferred between BMDCs, we isolated exosomes from *Wt* or *miR-146a−/−* BMDCs and administered them to *miR-146a−/−* BMDCs (Fig. 3c). Similar to miR-155, we observed that exosomal miR-146a was taken up by recipient BMDCs (Fig. 3d), and that miR-146a targets, IRAK1 and TRAF6, were repressed in recipient BMDCs receiving *Wt* but not *miR-146a−/−* exosomes looking at both the mRNA and protein levels (Fig. 3e–j). In addition, we calculated miR-146a copy number in *Wt* and *miR-146a−/−* exosomes where we found approximately one copy of miR-146a per exosome (Fig. 3k). miR-146a copy number was also calculated in *Wt* and *miR-146a−/−* donor BMDCs and BMDCs that received either *Wt* or *miR-146a−/−* exosomes (Fig. 3l). We observed an average of 370 copies present in recipient BMDCs following exosomes treatment. It has been suggested that 100–1,000 copies of miRNA per cell is likely to be functionally relevant^32^. As a control, we investigated the relative expression of miR-425, which was similar between the genotypes (Supplementary Fig. 3e,f). Our copy number data along with our observations of target knockdown are consistent with exosomally transferred miR-146a having functional relevance.

Recently, it has been shown in human B-cell lines that miRNAs are selectively packaged into exosomes based on 3′-non-templated nucleotide additions (NTAs)^17^ where 3′-uridylation was enriched in miRNAs contained in exosomes and 3′-adenylation was enriched in miRNAs retained in cells. To address whether we observe a similar phenomenon, RNA sequencing was performed using RNA from *Wt* donor BMDCs and *miR-155* and *miR-146a DKO* BMDCs that had received *Wt* exosomes. Next, we used a previously reported approach to identify NTAs in our data set^17^. However, we did not observe an enrichment of 3′-uridylation in transferred miR-155 or miR-146a (Supplementary Fig. 4). This difference from previous findings could be due to species and cellular differences (mouse primary BMDCs versus human B-cell lines) or further processing of the transferred miRNAs in recipient cells. However, we did observe some differences at certain nucleotide positions in each respective mature miRNA sequence when comparing *Wt* donor BMDCs with *DKO* BMDCs that received *Wt* exosomes (Supplementary Tables 1 and 2). These results are consistent with the idea that alterations to the mature miRNA sequence may influence miRNA loading into exosomes versus cellular retention.

### Seed-dependent repression of miRNA targets

**Figure 4:**
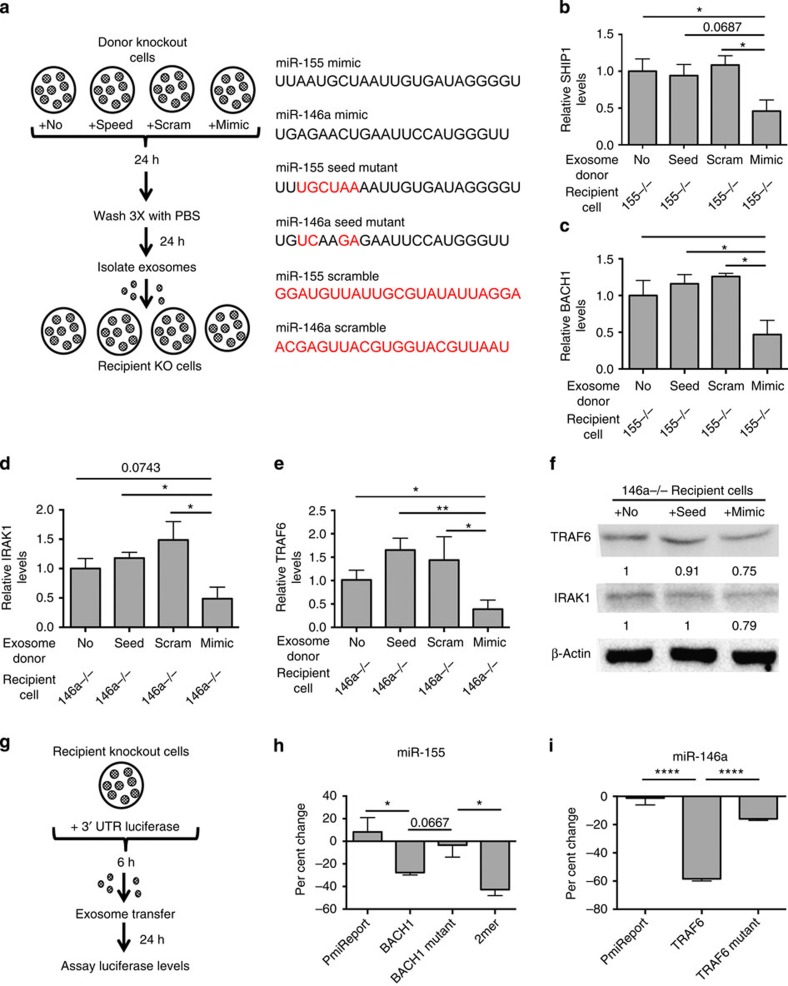
Seed-dependent repression of miRNA targets by exosome-delivered miR-155 and miR-146a. (**a**) Schematic for mimic experiment. (**b**,**c**) Relative mRNA levels of the miR-155 targets SHIP1 and BACH1 were measured via qRT–PCR in recipient cells that received exosomes with no mimics (No) (*n*=7), miR-155 seed mutant mimics (Seed) (*n*=4), with scrambled mimics (Scram) (*n*=3), or with miR-155-mimics (Mimic) (*n*=7). (**d**,**e**) qRT–PCR was preformed to assay the mRNA levels of the miR-146a targets, IRAK1 and TRAF6, following treatment with exosomes containing miR-146a mimics and controls as in **b**,**c**. Results are reported normalized to exosomes with no mimics added, which is set as 1. (**f**) Protein levels of TRAF6, IRAK1 and β-actin were determined via western blotting using lysates from *miR-146a−/−* BMDCs that received exosomes containing no mimics, seed mutant mimics or *Wt* mimics. Numbers below the blot represent relative protein levels with no mimics set as 1 following normalization to β-actin. (**g**) Schematic for 3′-UTR luciferase reporter assays in **h**,**i**. (**h**) Results from 3′-UTR luciferase reporter assays where *miR-155−/−* BMDCs were transfected with a pmiReport control vector, a BACH1 3′-UTR vector (BACH1), a BACH1 miR-155-binding site (bs) mutant vector (BACH1 mutant), or a 2mer-positive control vector. Transfected BMDCs were treated 6 h later with or without *Wt* exosomes and per cent change in luciferase activity of exosome treated BMDCs compared with no exosome treatment was calculated after 24 h (*n*=4). (**i**) Results from 3′-UTR luciferase reporter assay where *miR-146a−/−* BMDCs were transfected with a pmiReport control vector, a TRAF6 3′-UTR vector (TRAF6) or TRAF6 miR-146a bs mutant vector (TRAF6 mutant). Six hours later, the BMDCs were treated with or without *Wt* exosomes and per cent repression of luciferase activity was calculated 24 h after exosome transfer (*n*=4). Results represent two independent experiments. All data are presented as the mean±s.d. (error bars). *P<0.05; **P<0.01, ****P<0.0001; Student's *t*-test.

We next determined whether miR-155 and miR-146a mimic-loaded exosomes were sufficient to mediate direct target knockdown in recipient cells. *miR-155−/−* or *miR-146a−/−* BMDCs were transfected with either corresponding miRNA mimics, scrambled miRNA mimics, or seed mutant miRNA mimics for 24 h, then washed three times with PBS to remove any mimics that did not make it into the cells (Fig. 4a). We isolated exosomes from the cells after 24 h and transferred them to recipient knockout BMDCs. After 24 h, RNA was isolated from the cells and qRT–PCR was performed to assay the delivery of mimics and the knockdown of target mRNAs. We found that miRNA mimics could be successfully loaded into exosomes and delivered to recipient cells (Supplementary Figs 5 and 6). The transfer of miRNA mimics containing exosomes resulted in knockdown of respective target mRNAs in recipient BMDCs (Fig. 4b–f). However, exosomes that did not carry mimics, or that carried scrambled or seed mutant mimics, caused no change in target mRNA expression in recipient cells. These results indicate that exosomal miRNAs are responsible for direct target repression and are able to complement the target knockdown phenotype.

To further assess whether exosomal miRNA target repression was direct, we used 3′-untranslated region (UTR) luciferase reporter assays. Knockout BMDCs were transfected with 3′-UTR luciferase reporters for 6 h followed by treatment with or without *Wt* exosomes (Fig. 4g). *miR-155−/−* BMDCs were transfected with either a pmiReport empty vector control, BACH1 3′-UTR, BACH1 miR-155-binding site (bs) mutant 3′-UTR, or a miR-155-positive control (2mer). Luciferase activity in cells receiving the BACH1 3′-UTR or 2mer reporter constructs was reduced in response to miRNAs delivered by exosomes, while the exosomal miRNAs had little impact on luciferase activity in cells receiving the pmiReport empty vector or the BACH1 miR-155 bs mutant 3′-UTR reporter (Fig. 4h). In a separate experiment, *miR-146a−/−* BMDCs were transfected with either a pmiReport empty vector control, TRAF6 3′-UTR, or a TRAF6 miR-146a bs mutant 3′-UTR. The BMDCs transfected with the TRAF6 3′-UTR had decreased luciferase activity compared with the pmiReport empty vector and the TRAF6 miR-146a bs mutant 3′-UTR following exosome delivery of miRNAs (Fig. 4i). These results indicate that exosomally transferred miRNAs directly repress their targets via direct 3′-UTR interactions.

### Exosomal miR-155 and miR-146a modulate the response to LPS

**Figure 5:**
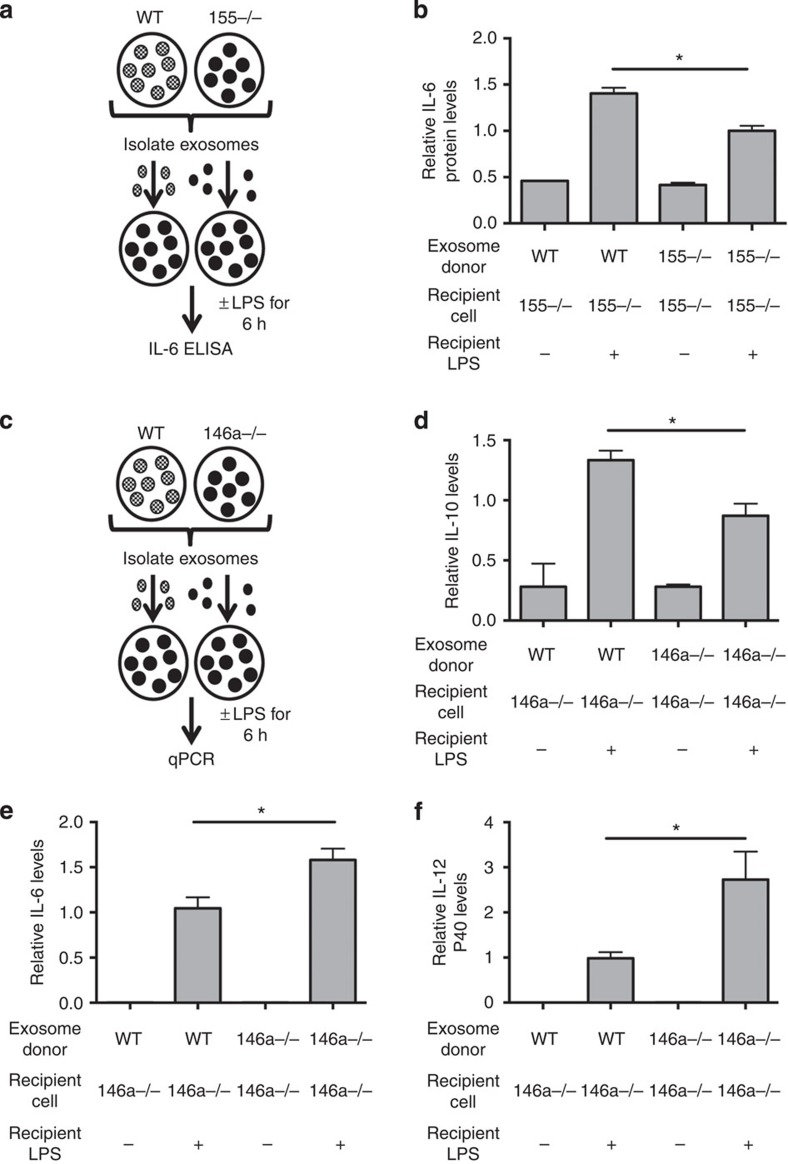
Exosomal transfer of miR-155 and miR-146a programme the response to LPS *in vitro* (**a**) A schematic of the experimental design for **b**. (**b**) Exosomes were isolated from *Wt* or *miR-155−/−* BMDCs and given to *miR-155−/−* BMDCs for 24 h. Cells were then treated with or without LPS and media was taken after 6 h for an IL-6 enzyme-linked immunosorbent assay. Relative IL-6 protein levels are shown (*n*=4). (**c**) Schematic for experiments in **d**–**f**. (**d**–**f**) qRT–PCR was used to quantify mRNA levels of IL-10, IL-6 and IL-12 p40 in *miR-146a−/−* BMDCs given exosomes from *Wt* or *miR-146a−/−* BMDCs for 24 h followed by stimulation with or without LPS for 6 h (*n*=4). Data represent two independent experiments. All data are presented as the mean±s.d. (error bars). *P<0.05; Student's *t*-test.

To determine whether the transfer of miR-155 via exosomes could affect the BMDC response to LPS, exosomes were isolated from *Wt* or *miR-155−/−* BMDCs and transferred to *miR-155−/−* BMDCs. Twenty-four hours later, cells were treated with LPS for 6 h (Fig. 5a). Consistent with a previously reported role for miR-155 in promoting interleukin (*IL*)*-6* expression^33^ and previously reported roles of miR-155-regulated responses to endotoxin^34^, cells that were treated with miR-155-containing exosomes produced more IL-6 on treatment with LPS for 6 h than cells having received *miR-155−/−* exosomes (Fig. 5b). These findings indicate that exosomes containing miR-155 can reprogramme recipient BMDCs in a manner that enhances their response to LPS.

miR-146a is known to induce an anti-inflammatory response to LPS^26^. Therefore, we wanted to investigate whether exosomally transferred miR-146a can programme BMDCs to respond in an anti-inflammatory manner, using a similar experimental setup as we did for miR-155 (Fig. 5c). BMDCs pre-treated with *Wt* exosomes produced more IL-10, but less IL-6 and IL-12 p40, following LPS stimulation than BMDCs that received *miR-146a−/−* exosomes (Fig. 5d–f). This gene expression profile demonstrates that miR-146a-containing exosomes reduce the pro-inflammatory response by BMDCs following LPS treatment. Without LPS treatment, there was no significant difference in *IL-10*, *IL-6* or *IL-12 p40* expression by cells receiving *Wt* versus *miR-146a−/−* exosomes (Fig. 5d–f), indicating that exosomal miR-146a specifically alters how these cells respond to LPS. Similar to miR-155, our data indicate that miR-146a is functionally transferred in exosomes and able to cause physiological changes in recipient cells. However, unlike miR-155, exosomal miR-146a acts to dampen the inflammatory response to LPS. These results are consistent with previous observations that miR-155 and miR-146a play opposing roles during inflammation^25^,^31^.

### miR-155 is transferred between immune cells *in vivo*

**Figure 6:**
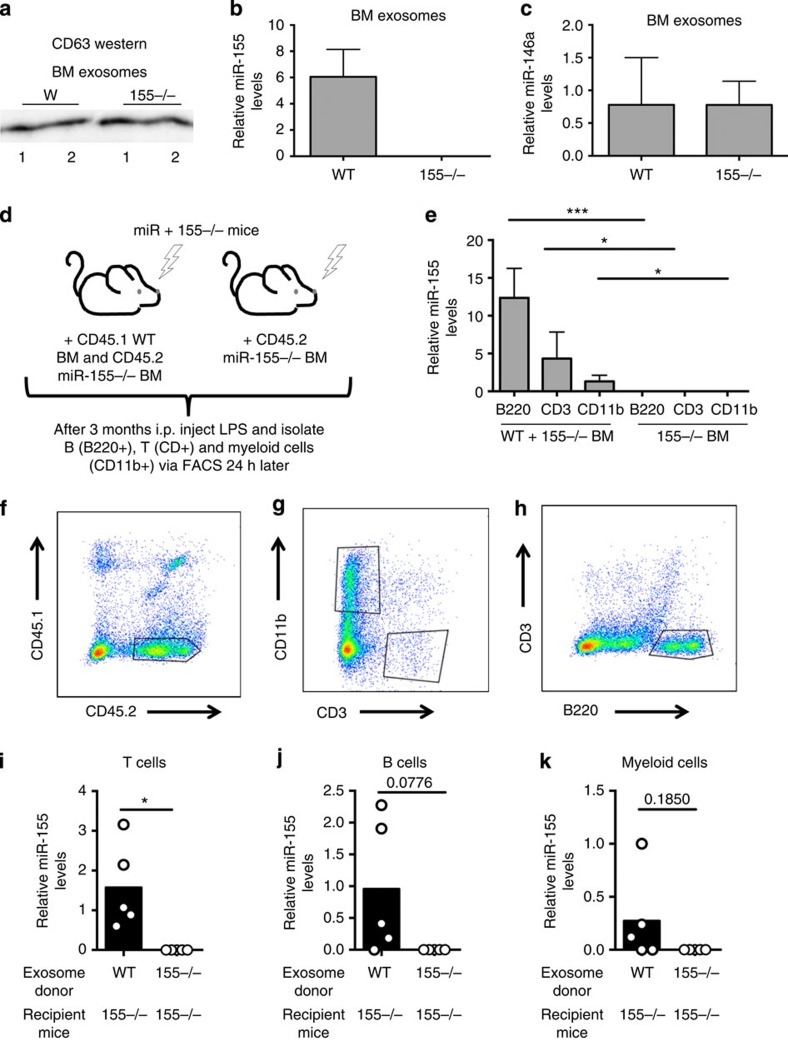
Transfer of endogenous miR-155 between haematopoietic cells *in vivo*. (**a**) CD63 western blotting using exosomes isolated directly from the BM of *Wt* or *miR-155−/−* mice. 1 and 2 stand for two biological replicates. (**b**,**c**) Levels of miR-155 and miR-146a in exosomes isolated form *Wt* and *miR-155−/−* mouse BM as measured by qRT–PCR (*n*=2). (**d**) Schematic of the *in-vivo* experiment. (**e**) qRT–PCR was used to quantify levels of miR-155 in *miR-155−/−* CD45.2^+^ BM cells that were B220^+^, CD3^+^, or CD11b^+^ from *miR-155−/−* mice that were either reconstituted with *Wt* (*CD45.1*^+^) and *miR-155−/−* BM, or *miR-155−/−* BM alone as indicated (*n*=5). (**f**–**h**) Representative FACS plots of the cell types in isolated BM shown in **e** (*n*=5). (**i**–**k**) *miR-155−/−* mice were i.p. injected multiple times over a week with either *Wt* or *miR-155−/−* exosomes. CD3^+^ T cells, B220^+^ B cells and CD11b^+^ myeloid cells were sorted from mouse spleens and qRT–PCR was preformed to analyse the delivery of miR-155 to each cell type (*n*=5). All data are presented as the mean±s.d. (error bars). *P<0.05; **P<0.01, ***P<0.001, Student's *t*-test.

As we observed functional transfer of miRNAs *in vitro*, we wanted to determine whether miRNAs could be transferred between immune cells *in vivo.* We first investigated whether exosomes were present in mouse BM by isolating exosomes directly from the BM of *Wt* and *miR-155−/−* mice using differential centrifugation. We found that both genotypes had exosomes present in the BM (Fig. 6a), and that miR-155 was expressed in *Wt* BM exosomes (Fig. 6b), whereas miR-146a was present in exosomes from both genotypes (Fig. 6c). We next determined whether miR-155 was passed between immune cells *in vivo.* To investigate this, *miR-155−/−* mice were lethally irradiated and reconstituted with either an equal mix of *CD45.1*^*+*^ *Wt* and *CD45.2*^*+*^ *miR-155−/−* BM or just miR-155−/− BM. After allowing 3 months for reconstitution, we injected mice with LPS to stimulate production of miR-155 by BM cells (Fig. 6d). BM cells were isolated 24 h after LPS stimulation and *miR-155−/−* haematopoietic cells were sorted via FACS according to their different CD45 alleles (Fig. 6f). We also further fractionated the *miR-155−/−* BM into B-cell, myeloid cell and T-cell fractions using the surface markers B220, CD11b and CD3, respectively (Fig. 6g,h). Using qRT–PCR, we detected miR-155 expression in *miR-155−/−* B cells, T cells and myeloid cells taken from *miR-155−/−* mice that had been reconstituted with both *Wt* and *miR-155−/−* BM (Fig. 6e). As a control, no miR-155 expression was observed in cells from mice reconstituted with only *miR-155−/−* BM. These data provide evidence that miR-155 is located within exosomes within the BM and is transferred between immune cells *in vivo*.

To determine whether miRNA-containing exosomes could deliver miRNAs to various cell types *in vivo*, we intraperitoneally (i.p.) injected ∼10^9^ exosomes derived from *miR-155−/−* or *Wt* BMDCs into *miR-155−/−* mice. After multiple injections over a week, the spleens of these mice were harvested and CD3^+^ T cells, B220^+^ B cells and CD11b^+^ myeloid cells were sorted via FACS. We found that miR-155 was delivered to all three of these cell types in the spleen (Fig. 6i–k). This indicates that exosomes are able to deliver miRNAs to various immune cell types.

### Exosomal miR-155 enhances inflammatory responses *in vivo*

**Figure 7:**
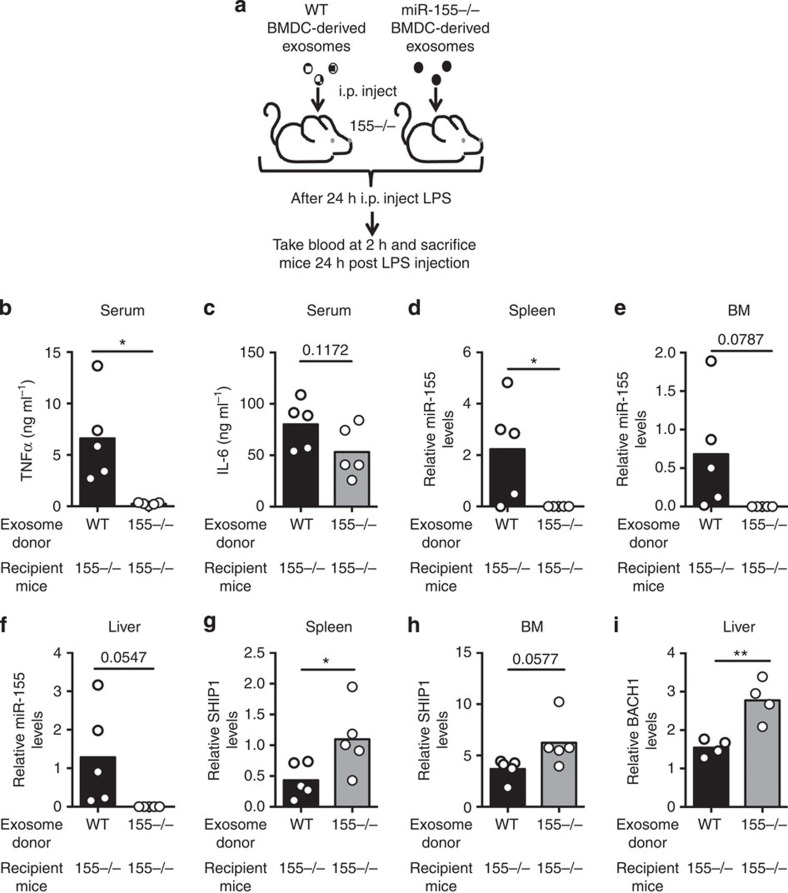
miR-155-containing exosomes promote a heightened response to LPS in *miR-155−/−* mice. (**a**) Schematic of the experimental design where *miR-155−/−* mice were i.p. injected with either *Wt* or *miR-155−/−* BMDC-derived exosomes and then challenged with LPS 24 h later. Blood was taken 2 h post LPS injection and the spleen, liver and BM were harvested 24 h post injection. (**b**,**c**) Serum TNFα and IL-6 concentrations were analysed via enzyme-linked immunosorbent assay 2 h after injection of LPS in *miR-155−/−* mice that had been pretreated with either *Wt* or *miR-155−/−* exosomes (*n*=5). (**d**–**f**) qRT–PCR was preformed using RNA isolated from the spleen, liver and BM, to assay the relative levels of exosomally delivered miR-155 (*n*=5). (**g**–**i**) mRNA levels of the miR-155 targets SHIP1 and BACH1 were measured in the spleen, liver and/or the BM using qRT–PCR (*n*=5). All data are presented as the mean±s.d. (error bars). *P<0.05; **P<0.01, Student's *t*-test.

Our *in vitro* data suggest that exosomally delivered miR-155 can increase the BMDC response to LPS (Fig. 5b). Owing to these observations, we investigated whether we could see the same effect *in vivo.* Approximately 10^9^ *Wt* or *miR-155−/−* BMDC-derived exosomes were i.p. injected into *miR-155−/−* mice, followed by administration of LPS 24 h later and collection of serum 2 h after that (Fig. 7a). The injection of *Wt* exosomes before LPS administration resulted in increased tumour necrosis factor-α (TNFα) and trending elevations in IL-6 serum concentrations compared with mice pretreated with *miR-155−/−* exosomes (Fig. 7b,c). In addition, we observed that miR-155 was delivered to the spleen, liver and BM, where we also found reduced target mRNA levels consistent with miR-155 activity in these tissues (Fig. 7d–i). These data demonstrate that miR-155 can be functionally delivered to a variety of tissues and cell types via exosome injection, and that this can increase the response to LPS *in vivo.*

### Exosomal miR-146a reduces inflammatory responses *in vivo*

**Figure 8:**
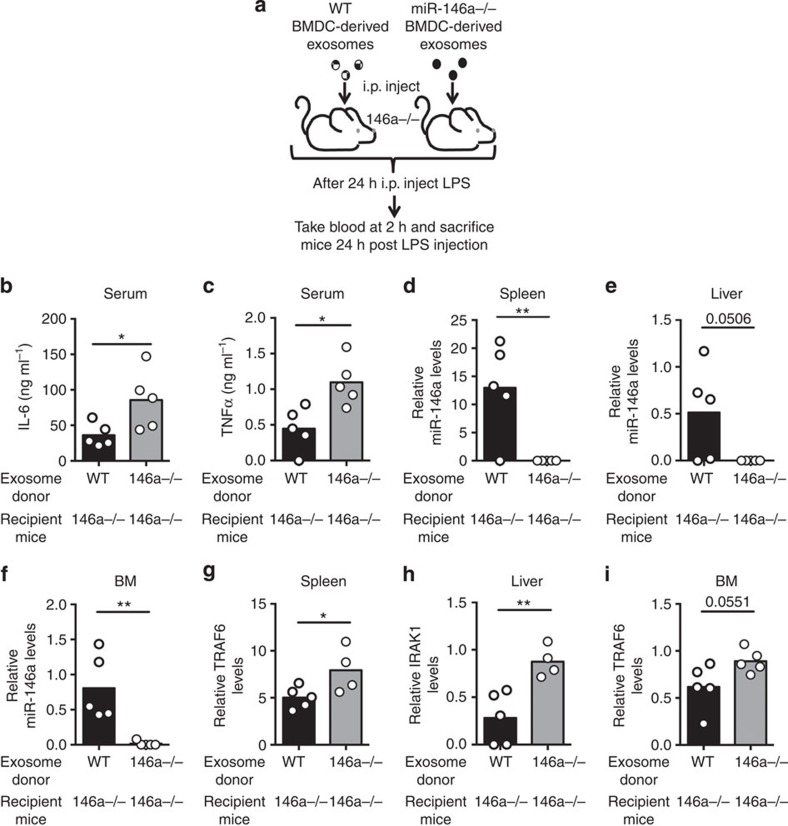
miR-146a-containing exosomes reduce inflammatory responses to LPS in *miR-146a−/−* mice. (**a**) Schematic of the experimental design where *miR-146a−/−* mice were i.p. injected with either *Wt* or *miR-146a−/−* BMDC-derived exosomes and then challenged with LPS 24 h later. Blood was taken 2 h post LPS injection and the spleen, liver and BM were harvested 24 h post injection. (**b**,**c**) Serum TNFα and IL-6 were analysed via enzyme-linked immunosorbent assay 2 h after injection of LPS (*n*=5). (**d**–**f**) qRT–PCR was preformed using RNA isolated from the spleen, liver and BM, to assay the relative levels of exosomally delivered miR-146a (*n*=5). (**g**–**i**) mRNA levels of the miR-146a targets TRAF6 and IRAK1 were measured in the spleen, liver and/or the BM using qRT–PCR (*n*=5). All data are presented as the mean±s.d. (error bars). *P<0.05; **P<0.01, Student's *t*-test.

**Figure 9:**
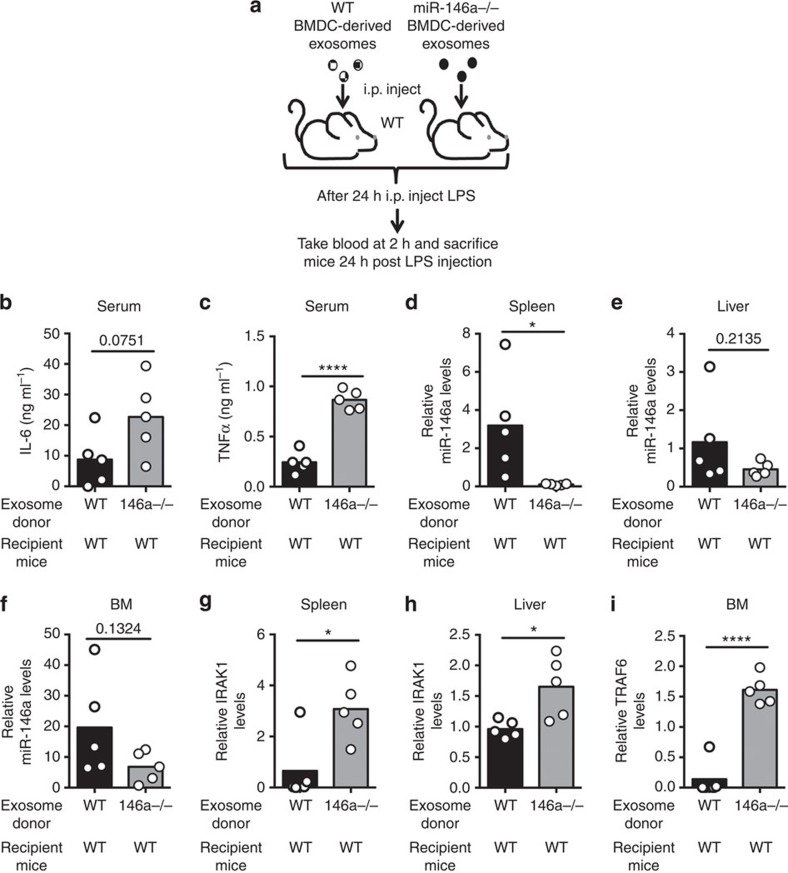
miR-146a-containing exosomes reduce inflammatory response to LPS in *Wt* mice. (**a**) Schematic of the experimental design where *Wt* mice were i.p. injected with either *Wt* or *miR-146a−/−* BMDC-derived exosomes and then challenged with LPS 24 h later. Blood was taken 2 h post LPS injection and the spleen, liver and BM were harvested 24 h post injection. (**b**,**c**) Serum TNFα and IL-6 were analysed via enzyme-linked immunosorbent assay 2 h after injection of LPS (*n*=5). (**d**–**f**) qRT–PCR using RNA isolated from the spleen, liver and BM was performed to assay the relative levels of exosomally delivered miR-146a (*n*=5). (**g**–**i**) mRNA levels of the miR-146a targets TRAF6 and IRAK1 were measured in the spleen, liver and/or the BM using qRT–PCR (*n*=5). Results represent two independent experiments. All data are presented as the mean±s.d. (error bars). *P<0.05; ****P<0.0001, Student's *t*-test.

We next investigated whether exosomes containing miR-146a would have an anti-inflammatory impact following LPS administration to mice. Approximately 10^9^ exosomes were isolated from *Wt* or *miR-146a−/−* BMDCs and injected i.p. into *miR-146a−/−* mice. Twenty-four hours later, the mice were given LPS and serum was collected after 2 h to assay inflammatory cytokine levels (Fig. 8a). Mice having received *Wt*, miR-146a-containing exosomes had reduced TNFα and IL-6 serum concentrations after LPS administration compared with mice having received miR-146a-deficient exosomes (Fig. 8b,c). Exosomes alone had a negligible effect on cytokine levels *in vivo*. Further, 24 h after LPS injection we isolated the spleen, liver and BM, and found that miR-146a was clearly present in these tissues from mice receiving *Wt* exosomes but was not present in tissues from mice that received *miR-146a−/−* exosomes (Fig. 8d–f). In addition, miR-146a targets involved in Toll-like receptor (TLR) signalling were repressed in tissues in mice that received *Wt* exosomes (Fig. 8g–i). Similar results were obtained when miR-146a-containing exosomes were administered to *Wt* recipients (Fig. 9a–i). Together, these data demonstrate that exosomal miR-146a can reduce the inflammatory response to LPS in mice.

## Discussion

Although exosomes have been studied for a number of years, the biological roles of exosomal miRNAs are just beginning to be investigated^4^,^5^,^6^,^7^,^8^. Our data demonstrate that miRNAs 155 and 146a are released from BMDCs in exosomes, are taken up by recipient BMDCs and subsequently mediate target gene repression. In addition, we have found that the transfer of miR-155 or miR-146a can alter the ability of recipient cells to respond to inflammatory cues both *in vitro* and *in vivo*. The capacity of these transferred miRNAs to influence the response of BMDCs to a pro-inflammatory stimulus suggests that the transfer of miRNAs is an important mechanism by which immune cells are primed to respond to an imminent encounter with a microbe. As miR-155 and miR-146a have been shown to regulate inflammation in a variety of contexts, our findings provide novel insights into how and where they function, providing a greater understanding of how they regulate mammalian immunity. Furthermore, our study adds to the growing body of evidence that miRNA transfer within exosomes is part of the intercellular communication networks that coordinates complex immune responses^8^,^12^,^14^.

Previous studies have used cell lines, miRNA overexpression and/or miRNA reporter constructs to study exosomal transfer of miRNAs^12^,^13^,^14^. Although these approaches have provided important evidence that miRNAs can be transferred in exosomes, we designed our approach to be as physiologically relevant as possible. miRNAs were produced at endogenous levels by primary cells and established endogenous miRNA target genes were used as readouts for miRNA activity in recipient cells. Furthermore, exosomes were purified away from other BMDC factors, such as cytokines, and miR-155- and miR-146a-deficient recipient cells were used to confidently track the delivery and specific effects of the exosomally delivered miRNA both *in vitro* and *in vivo*.

A recent report has claimed that the amounts of specific miRNAs contained within exosomes is less than one copy per exosome^35^. Our copy number analysis found there to be approximately one copy of miR-146a per exosome, consistent with exosomes having low content of individual miRNAs. However, we found that one BMDC produces ∼500 exosomes after 24 h of culture, indicating that each cell is able to release at least hundreds of copies of miR-146a in exosomes to be delivered to recipient BMDCs and mediate target knockdown. Thus, it seems that the large numbers of exosomes produced per cell allows for the loading of low miRNA numbers per exosome to achieve functional relevance.

It is important to note that exosome populations produced by *Wt* cells contain both miR-155 and miR-146a, which we show have either pro- or anti-inflammatory effects, respectively. There are several possible reasons why exosome populations would contain both of these functionally distinct miRNAs species. First, exosomes could be transferring both pro- and anti-inflammatory miRNAs together to buffer inflammatory responses by recipient cells, to achieve the optimal magnitude of response. Second, it is plausible that miR-155 and miR-146a are located in separate exosomes that are delivered to different target cell types. A third possibility is that miR-155 and miR-146a release in exosomes is a dynamically regulated process where the ratio of miR-155 to miR-146a changes over time. For example, immune cells that have sensed a pathogen could initially release exosomes with high levels of pro-inflammatory miRNAs such as miR-155 followed by a shift to anti-inflammatory miRNAs such as miR-146a during the resolution phase of the response. These possibilities will be explored in future studies wherein the analysis of single exosomes may be required.

Exosomes are clearly complex vesicles that contain an assortment of different membrane and soluble proteins, as well as different types of RNAs, including miRNAs^3^. Thus, we cannot formally rule out that exosomes produced by *Wt* versus *miR-155−/−* or *miR-146a−/−* BMDCs may differ in some aspect other than the presence or the absence of the corresponding miRNA that has been genetically deleted, and that this may also have some influence on the inflammatory response by recipient cells. However, we have been able to address this possibility to some degree by successfully complementing the exosomal miRNA target gene phenotypes by loading miRNA mimics into miRNA knockout exosomes. Further, we were also able to demonstrate that target repression is direct through the use of seed mutant mimics that failed to repress target gene expression in recipient cells as well as 3′-UTR luciferase reporter assays where binding-site mutant 3′-UTRs were not repressed by exosomally transferred miRNAs. Collectively, these data strongly support the idea that individual miRNAs in exosomes are transferred between cells in a functionally relevant manner.

We also observed transfer of miRNAs between immune cells *in vivo*, indicating that this mechanism of cellular communication is also occurring in a physiologically relevant setting. Future work will be needed to isolate and study distinct cell types in the context of exosome production and uptake, as tissues such as the spleen, liver and BM are made up of a heterogeneous populations of cells that probably differ in their capacity to participate in these processes. Further, we predict that functional miRNA transfer via exosomes will be most relevant in defined microenvironments such as stem cell niches or within tumours, where exosome concentrations might be at their highest.

Future studies will also require reagents where the production of miRNA-containing exosomes can be specifically blocked *in vivo* to assess the relevance of this mechanism in distinct inflammatory settings. *Rab27 DKO* mice will provide one such reagent despite possible roles for Rab27 a and b in exosome-independent cellular processes. However, it is unclear whether the release of pro- or anti-inflammatory exosomal miRNAs will have a dominant impact on the inflammatory response *in vivo*, and we predict that this will probably be context dependent. However, as *Rab27 DKO* regulatory T cells have recently been shown to be functionally impaired, it is likely to be that these animals will have heightened inflammatory responses^23^. Consequently, the relevance of exosomal miRNA release by distinct immune cell types may have to be studied using cell-type-specific *Rab27 DKO* mice.

Exosomal miRNAs are currently being extensively studied as biomarkers of disease, as their serum levels are altered in a variety of pathological conditions^36^,^37^,^38^. Our results, in combination with others, suggest that these differences have functional consequences. As exosomes appear to be a natural way that cells transfer miRNAs, there is also growing interest in understanding the therapeutic potential of exosomes as delivery vehicles for specific miRNAs or their inhibitors^39^,^40^,^41^,^42^,^43^,^44^. Producing exosomes from patients' own cells may serve as an ideal vehicle for autologous therapies involving miRNA delivery, and our capacity to load miRNA mimics suggests that the miRNA content of exosomes can be manipulated. Further, we clearly demonstrate that injection of miR-146a- and miR-155-containing exosomes results in delivery of these miRNAs to a variety of mouse tissues, repression of target genes and an altered inflammatory response *in vivo*, where miR-155 promoted and miR-146a repressed inflammation in response to endotoxin. This suggests that exosomal miR-146a could be used as a prophylaxis or therapy to treat inflammatory diseases, such as bacterial sepsis. Conversely, exosomal miR-155 could be used as an adjuvant to improve vaccine efficacy. However, it is also clear that the full spectrum of applications whereby exosomal miRNAs can be used is potentially quite broad and will require a great deal of future work. As we refine our understanding of how miRNAs are loaded into exosomes and delivered in a functional manner to specific recipient cells, such therapeutic approaches may become feasible in the clinic.

## Methods

### Mice

*miR-155−/−* (Allan Bradley Lab, Sanger Institute), *miR-146a−/−* (David Baltimore Lab, California Institute of Technology), *miR-155* and *miR-146a DKO* (Ryan O'Connell, University of Utah), *Wt* (Jackson Labs) and *CD45.1 Wt* (Jackson Labs) are on a C57BL6 genetic background and housed in the animal facility at the University of Utah. *Rab27 DKO* (Rab27a ash/ash Rab27b−/−) mice (Tanya Tolmachova and Miguel C. Seabra, Imperial College London) were housed at the Imperial College London under the UK Home Office animal project license 70/7078 and the BM was sent to Utah for experiments together with the BM from the *Wt* animals of similar age, sex and background (C57BL6). Experiments were approved by the Institutional Animal Care and Use Committee at the University of Utah. Mice were age matched and sex matched, and were in the age range of 8–16 weeks old. For BM reconstitutions, lethal irradiation (1,000 rads) was delivered using an X-ray source. Following irradiation, mice were injected with three million BM cells via retro-orbital injection. *Escherichia coli* LPS (Sigma) was administered through i.p. injections at a sub-lethal concentration of 50 μg^29^. In other experiments, exosomes were i.p. injected 24 h before LPS injection of the same concentration.

### Cells culture

BMDCs were derived from mouse BM by culturing red blood cell-depleted BM in complete RPMI (10% fetal bovine serum, 100 units per ml penicillin and 100 units per ml streptomycin, β-mercaptoethanol, glutamate, sodium pyruvate, HEPES and non-essential amino acids) with 20 ng ml^−1^ granulocyte machrophage colony-stimulating factor for 3–4 days at 37 °C with 5% CO_2_. The cells were then cultured in 5 ml complete RPMI with 20 ng ml^−1^ granulocyte machrophage colony-stimulating factor for an additional 3–4 days for a total of 7 days in culture. LPS stimulation was performed at a concentration of 500 ng ml^−1^. Cells were separated using a Transwell Permeable Support 0.4 μm Polycarbonate Membrane 24 mm insert six-well plates (Costar).

### RNA sequencing

*Wt* exosomes were transferred to recipient *miR-155* and *miR-146a DKO BMDCs*. Three biological replicates from *Wt* donor and exosome-recipient *DKO* BMDCs were submitted to the University of Utah's High Throughput Genomic Core for Illumina TrueSeq Small RNA Sample Prep. NTAs were identified and frequencies of A, G, C and U additions were calculated as described previously^17^ by our bioinformatics core facility. In addition, we analysed each position in the mature miRNA sequences of miR-155 and miR-146a, and calculated the percentage of observed bases at each position to determine any changes in nucleotide composition between miRNAs in donor versus exosome-recipient BMDCs. RNA sequencing data are deposited in GEO with the accession number GSE67946.

### Copy-number analysis

miRNA copy number was calculated in *Wt* and *miR-146a−/−* donor cells, exosomes and *miR-146a−/−* BMDCs that received *Wt* exosomes. Total RNA was isolated (using the miRNeasy kit) from one million donor BMDCs and one million recipient BMDCs that were cultured with exosomes from one million donor BMDCs collected after 24 h or exosomes isolated from one million BMDCs after 24 h. Thirty nanograms of RNA isolated from these samples was then used for qRT–PCR analysis. To make a standard curve, 1 ng of synthetic single-stranded miR-146a (IDT custom RNA oligo—sequence: 5′-UGAGAACUGAAUUCCAUGGGUU-3′) was spiked into either one million *miR-146a−/−* BMDCs or exosomes isolated from one million *miR-146a−/−* BMDCs, and total RNA was isolated in the same manner as our experimental samples (miRNeasy). Thirty nanograms of this isolated RNA was used to preform a complementary DNA reaction to use for standard curves. Standard curves for cells and exosomes were made with these cDNA samples via serial dilutions and cp values were determined via qPCR with miR-146a primers. The BMDC standard curve was then used to determine copy number in our cellular samples and the BMDC exosome standard curve was used to determine the copy number of miR-146a in our exosome samples.

### Mimic

miRNA mimics were purchased from Qiagen. Scrambled, seed mutant and miR-mimic sequences are as follows:

miR-146a scramble (5′-ACGAGUUACGUGGUACGUUAAU-3′),

miR-146a seed mutant (5′-UGUCAAGAGAAUUCCAUGGGUU-3′),

miR-146a mimic (5′-UGAGAACUGAAUUCCAUGGGUU-3′),

miR-155 scramble (5′-GGAUGUUAUUGCGUAUAUUAGGA-3′),

miR-155 seed mutant (5′-UUUGCUAAAAUUGUGAUAGGGGU-3′) and

miR-155 mimic (5′-UUAAUGCUAAUUGUGAUAGGGGU-3′). Donor cells were transfected with 30 μl of the hi-perfect transfection reagent (Qiagen) in 2 ml of serum-free media with 60 ng of each mimic. After 24 h, cells were washed three times with PBS and given fresh medium. Exosomes were isolated 24 h after washing and transferred to recipient cells for 24 h.

### Luciferase reporter assay

Knockout BMDCs (2.5 × 10^5^) were transfected with 3′-UTR luciferase reporter constructs (for *mir-155−/−*: pmiReport, Bach1, Bach1 155 mutant, 2mer^29^; (for miR-146−/−: pmiReport, Traf6, Traf6 146a mutant^45^) using Lonza's Amaxa Mouse Dendritic Cell Nucleofector Kit, according to manufacturer's instructions. After 6 h of nucleofection, BMDCs were treated with or without *Wt* BMDC-derived exosomes and luciferase activity was measured 24 h later using a Dual Luciferase Kit (Promega). Luciferase repression of exosome-treated BMDCs compared with no exosome treatment was calculated and graphed as per cent change in luciferase activity. Renilla luciferase was used to normalize firefly luciferase values. 2 μg of each construct was transfected into BMDCs.

### Exosome isolation and procedures

For *in-vitro* experiments, we isolated exosomes from approximately one million BMDCs cultured in media for 24 h and transferred them to the same number of recipient BMDCs. Differential centrifugation was performed to isolate exosomes from conditioned medium. Initial spins consisted of a 10-min spin at 1,000*g*, a 2,000*g* spin for 10 min and a 10,000*g* spin for 30 min. The supernatant was retained each time. The supernatant was then spun at 100,000*g* for 70 min and the pellet was re-suspended in 1 × PBS, to dilute remaining soluble factors, followed by another centrifugation at 100,000*g* for 70 min. The final pellet contained the exosomes, which were re-suspended in tissue culture media. This protocol is based on previous exosome isolation methods^46^. We used either a Beckman ultracentrifuge with a TI75 fixed angle rotor or a Thermo Scientific Sorvall Lynx 6000 with a T26-8 × 50 rotor. GW4869 is a neutral sphingomyelinase 2 inhibitor that has been previously used to prevent exosome release^13^,^15^. In some experiments, we treated BMDCs with 10 μM GW4869 (Sigma-Aldrich) or vehicle for 24 h.

Exosome numbers for the miR-146a and miR-155 *in-vivo* experiments were determined using the EXOCET Exosome Quantification Assay Kit from System Biosciences, according to kit instructions. Three plates of approximately three million BMDCs each were cultured in media for 3 days. The supernatant from these plates was collected and exosomes were isolated as described above.

### Western blotting and enzyme-linked immunosorbent assay

Protein was isolated with RIPA lysis buffer (RIPA buffer, phenylmethyl sulfonyl fluoride, NaF, NaVO_4_ and protease inhibitor). Total protein levels were quantified using a Bio-Rad protein assay and equal amounts of protein were loaded and separated using 12% (TRAF6, Ago2 and CD63) or 8% (SHIP1 and IRAK1) SDS–PAGE followed by immunoblotting with appropriate antibodies. Antibodies include the following: α-TRAF6 at 1:500 dilution (EP591Y Abcam, ab33915), α-β-actin antibody at 1:1,000 dilution (mAbcam 8226, ab8226), α-Ago2/eIF2C2 antibody at 1:200 dilution (Abcam, ab32381), α-CD63 (H-193) at 1:200 dilution (Santa Cruz Biotechnology, sc-15363), α-SHIP1 (V-19) at 1:250 dilution (Santa Cruz Biotechnology, sc-1963), and α-IRAK1 D5167 at 1:500 Dilution (Cell Signaling, 4504). Western blottings were quantified using ImageJ software. The enzyme-linked immunosorbent assay used to quantify mouse IL-6 and TNFα concentrations were obtained from eBioscience and were performed using the manufacturer's suggested protocol. Images have been cropped for presentation. Full-size images are presented in Supplementary Figs 7–9.

### RNA isolation and qRT–PCR

RNA isolation was performed using Qiagen's miRNeasy kit, according to manufacturer's instructions. Mature miRNA cDNA was made with a miRCURY LNA universal RT miRNA PCR kit using 10 ng of RNA from each sample (Exiqon). qPCR of mature miRNA was performed with the miRCURY LNA universal RT miRNA PCR kit SYBR green master mix (Exiqon) with LNA primers for miR-146a-5p (Exiqon), mmu-miR155-5p (Exiqon), mmu-miR-425-5p (Exiqon) and 5s rRNA (Exiqon). Custom LNA primers were also made and designed by Exiqon to detect the miR-155 and miR-146a seed mutant mimics (miR-146a design ID 410833-1) (miR-155 design ID 410829-1). 5s was used to normalize expression. cDNA from total RNA was made with qScript using 30 ng of RNA from each sample (Quanta). qPCR was performed with Promega GoTaq pPCR master mix. Primer sequences are as follows:

SHIP1-F (5′-GAGCGGGATGAATCCAGTGG-3′),

SHIP1-R (5′-GGACCTCGGTTGGCAATGGTA-3′),

BACH1-F (5′-TGAGTGAGAGTGCGGTATTTGC-3′),

BACH1-R (5′-GTCAGTCTGGCCTACGATTCT-3′),

HO1-F (5′-TGACACCTGAGGTCAAGCAC-3′),

HO1-R (5′-TCCTCTGTCAGCATCACCTG-3′),

IRAK1-F (5′-TGTGCCGCTTCTACAAAGTG-3′),

IRAK1-R (5′-TGTGAACGAGGTCAGCTACG-3′),

TRAF6-F (5′-AAGCCTGCATCATCAAATCC-3′),

TRAF6-R (5′-CTGGCACTTCTGGAAAGGAC-3′).

L32-F (5′-AGCTCCCAAAAATAGACGCAC-3′) and

L32-R (5′-TTCATAGCAGTAGGCACAAAGG-3′). L32 levels were used to normalize mRNA expression levels.

### Electron microscopy

EM samples were prepared using differential centrifugation from BMDC-conditioned media. Exosomal pellets were re-suspended in PBS and processed by the University of Utah's EM core facility for cryo-EM analysis.

### Immunoprecipitations

An anti-pan Ago antibody (clone 2A8, Millipore) was was used to IP Ago proteins. α-AGO and IgG control coated beads were prepared by incubating magnetic protein G beads (Active motif) with each respective antibody in IP lysis buffer (0.5% NP40, 150 mM KCl, 1 mM NaF, 25 mM Tris, 2 mM EDTA, protease inhibitor and 0.5 mM dithiothreitol) with rotation overnight at 4 °C. One-third of the protein lysate prepared from BMDCs that had received either *Wt* or *miR-155−/−* exosomes using IP lysis buffer was used for the IP. Bead–antibody mixes were washed three times with lysis buffer with rotation at 4 °C, re-suspended in lysis buffer and added to the lysates. Lysates were incubated with bead–antibody mix at 4 °C with rotation overnight and then washed six times with IP wash buffer (300 mM NaCl, 50 mM Tris,.01% NP40, 5 mM MgCl_2_, 129 ml dH_2_O), with the last wash done using PBS. Protein was isolated from a fraction of the sample with 1 × Laemmli diluted in lysis buffer and RNA was isolated from another fraction using miRNeasy extraction. A western blotting for AGO2 (Abcam) and qRT–PCR analysis for miR-155 and miR-146a were performed to confirm AGO pulldown and association with these miRNAs.

### Flow cytometry

Fluorophore-conjugated monoclonal antibodies specific to CD45.1, CD45.2, B220, CD3, CD11b or CD11c (Biolegend) were used to stain red blood cell-depleted BM and spleen cells. These populations were sorted using a FACS Aria II in the Flow Cytometry Core Facility at the University of Utah.

### Statistics

Data were analysed using Student's *t*-tests, to determine statistically significant differences between relevant samples. *P*-values were either listed or represented by the following number of asterisks: *P<0.05; **P<0.01; ***P<0.001; ****P<0.0001.

## Additional information

**How to cite this article:** Alexander, M. *et al.* Exosome-delivered microRNAs modulate the inflammatory response to endotoxin. *Nat. Commun.* 6:7321 doi: 10.1038/ncomms8321 (2015).

## Supplementary Material

Supplementary Information Supplementary Figures 1-9 and Tables 1-2
