# Supplementary material for: Exosome-delivered microRNAs modulate the inflammatory response to endotoxin
Source: Nat Commun. 2015 Jun 18;6:7321. doi: 10.1038/ncomms8321 (PMC4557301; doi:10.1038/ncomms8321)
Supplement: Supplementary Information — Supplementary Figures 1-9 and Tables 1-2 [file ncomms8321-s1.pdf]

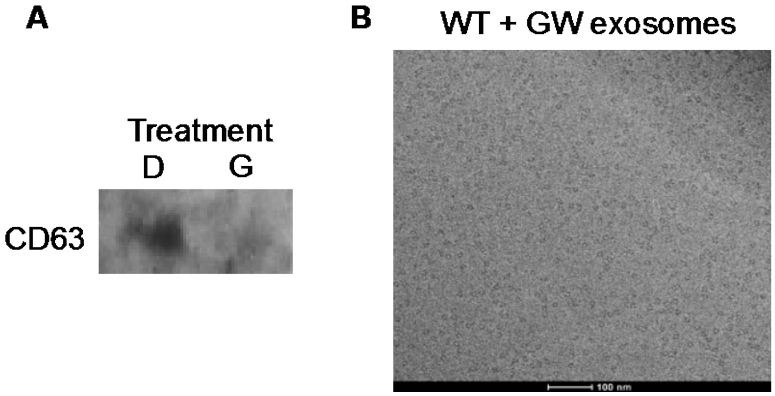

Supplemental Figure 1 - Exosome isolation from BMDCs treated with and without GW4869. (A) CD63 Western blot from the protein lysed exosome pellet isolated from BMDCs treated with GW4869 or DMSO vehicle control. **(B)** EM image of the exosome pellet from BMDCs treated with GW4869. Scale bar is 100nm.

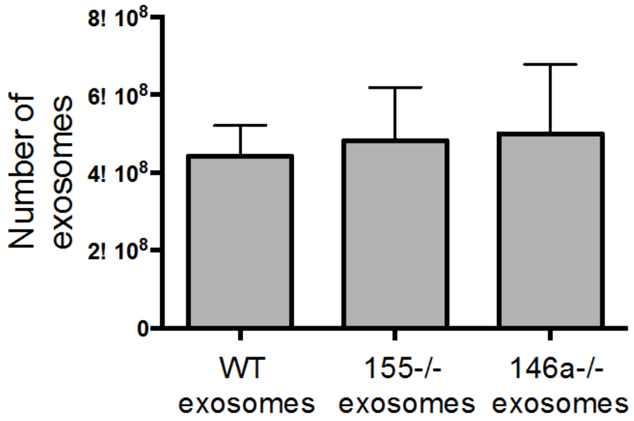

Supplemental Figure 2 - Quantification of exosomes produced by BMDCs. Exosomes were isolated from approximately 1 million miR-146a-/-, miR-155-/- or WT BMDCs that were cultured in media for 24 hours (n=3). Quantification was preformed with the EXOCET kit. Limit of detection is $2X10^{7}$ exosomes. All data are presented as the mean +/- S.D. (error bars).

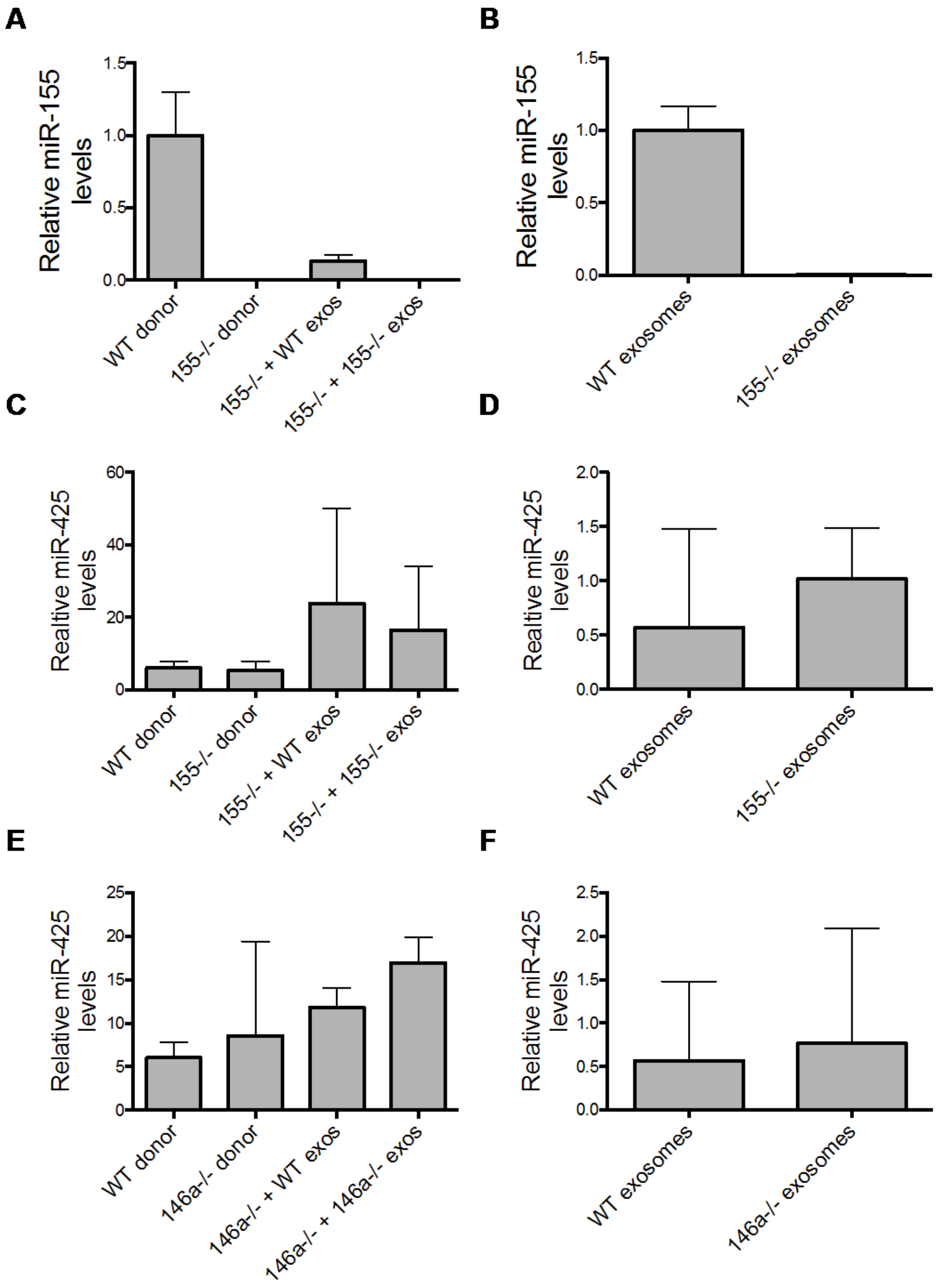

Supplemental Figure 3 - Relative expression of miR-155 and miR-425 in donor cells, exosomes and recipient cells. (**A**) Relative expression of miR-155 in Wt (Wt donor) and miR-155-/- (155-/- donor) donor BMDCs and miR-155-/- recipient BMDC that received either Wt (155-/- + Wt exos) or miR-155-/- (155-/- + 155-/- exo) exosomes (n=3). (**B**) Relative expression of miR-155 in miR-155-/- and Wt exosomes as determined by qRT-PCR (n=3). **(C-D)** Relative miR-425 levels were measured via qRT-PCR in same cells as in (A) and same exosomes as in (B) (n=3). **(E)** Relative miR-425 levels were measured via qRT-PCR in Wt (Wt donor) or miR-146a-/- BMDCs (146a-/- donor) and miR-146a-/- recipient BMDCs that received either Wt (146a-/- + Wt exos) or miR-146a-/- exosomes (146a-/- + 146a-/- exos) (n=3). **(F)** Relative miR-425 levels were measure via qRT-PCR in Wt and miR-146a-/- exosomes (n=3). All data are presented as the mean +/- S.D. (error bars).

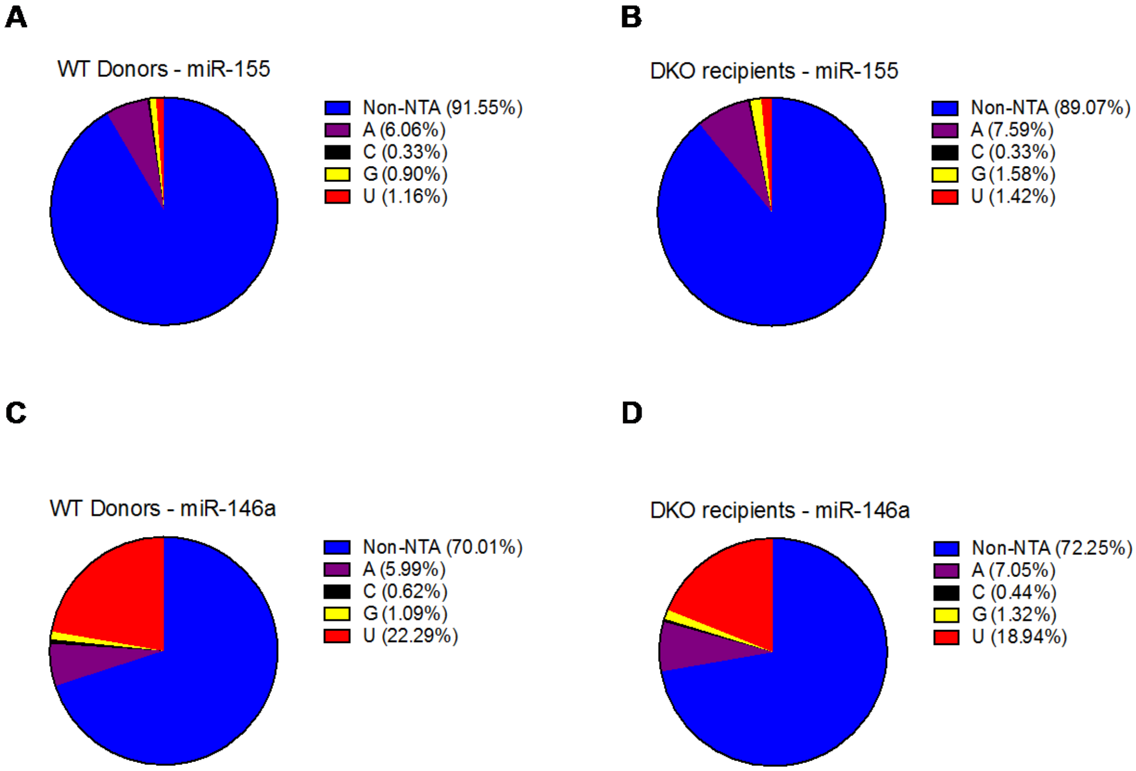

Supplemental Figure 4 – Mature miRNA sequence differences between donor and exosome treated recipient BMDCs. (A-B) Percentages of mature miR-155 without NTAs or with A, C, G and U NTAs were determined by RNA-Seq using RNA from Wt donor BMDCs and DKO BMDCs that received Wt exosomes (n=3). **(C-D)** Percentages of mature miR-146a non-NTA sequences as well as A, C, G and U NTAs were determined by RNA-Seq using RNA from Wt donor BMDCs and DKO BMDCs that received Wt exosomes (n=3).

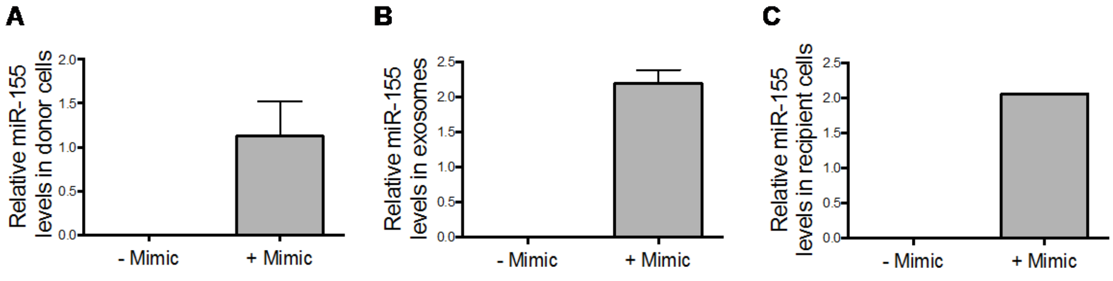

Supplemental Figure 5 - Transfected miRNA mimics are loaded into exosomes and transferred to recipient cells. (**A**) miR-155 mimics can be detected in miR-155-/- BMDCs after transfection for 24 hours (n=2). (**B**) miR-155 mimics can be detected in exosomes from miR-155-/- BMDCs transfected with mimics as assayed by qRT-PCR (n=2). **(C)** qRT-PCR was used to determine levels of miR-155 mimics in recipient miR-155-/- BMDCs given either miRNA loaded exosomes or exosomes lacking miR-155. All data are presented as the mean +/- S.D. (error bars).

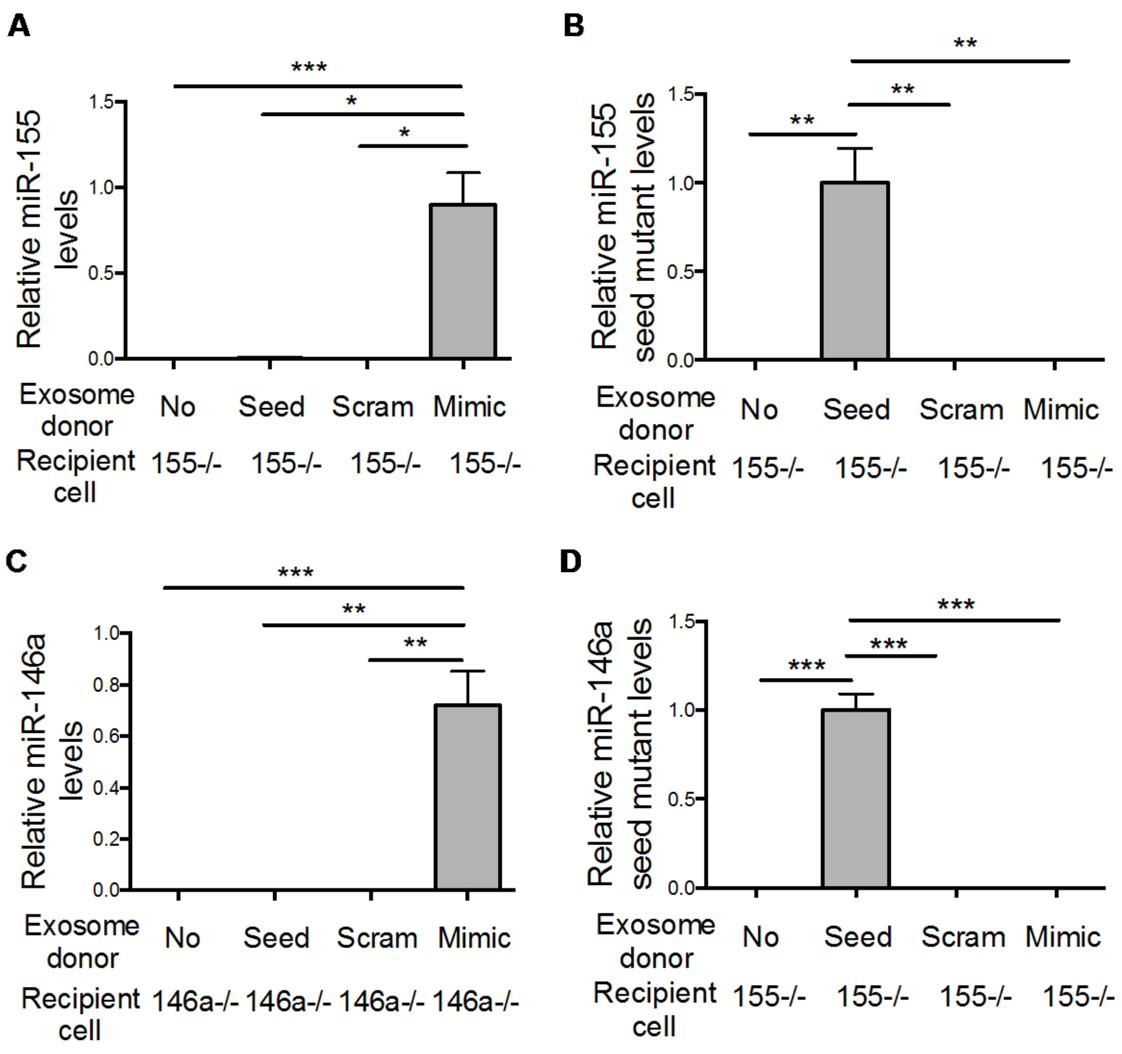

Supplemental Figure 6 – Exosomally delivered miRNA mimics and seed mutant mimics can be detected in recipient cells following delivery by exosomes. (A) Levels of miR-155 mimics were measured via qRT-PCR in recipient miR-155-/- BMDCs that had been given exosomes derived from donor miR-155-/- BMDCs transfected with either no mimics, seed mutant mimics, scrambled mimics or Wt miR-mimics (n=3). **(B)** Levels of seed mutant miR-155 mimics in the same cells as in (A). **(C)** Levels of miR-146a mimics were measured via qRT-PCR in miR-146a-/- recipient BMDCs that had been given exosomes derived from donor miR-146a-/- BMDCs transfected with either no mimics, seed mutant mimics, scrambled mimics or Wt miR-mimics (n=3). **(D)** Levels of seed mutant miR-146a mimics in the same cells as in (C). All data are presented as the mean +/- S.D. (error bars). *, $p < 0.05$; **, $p < 0.01$, *** $P < 0.001$, Student's t-Test.

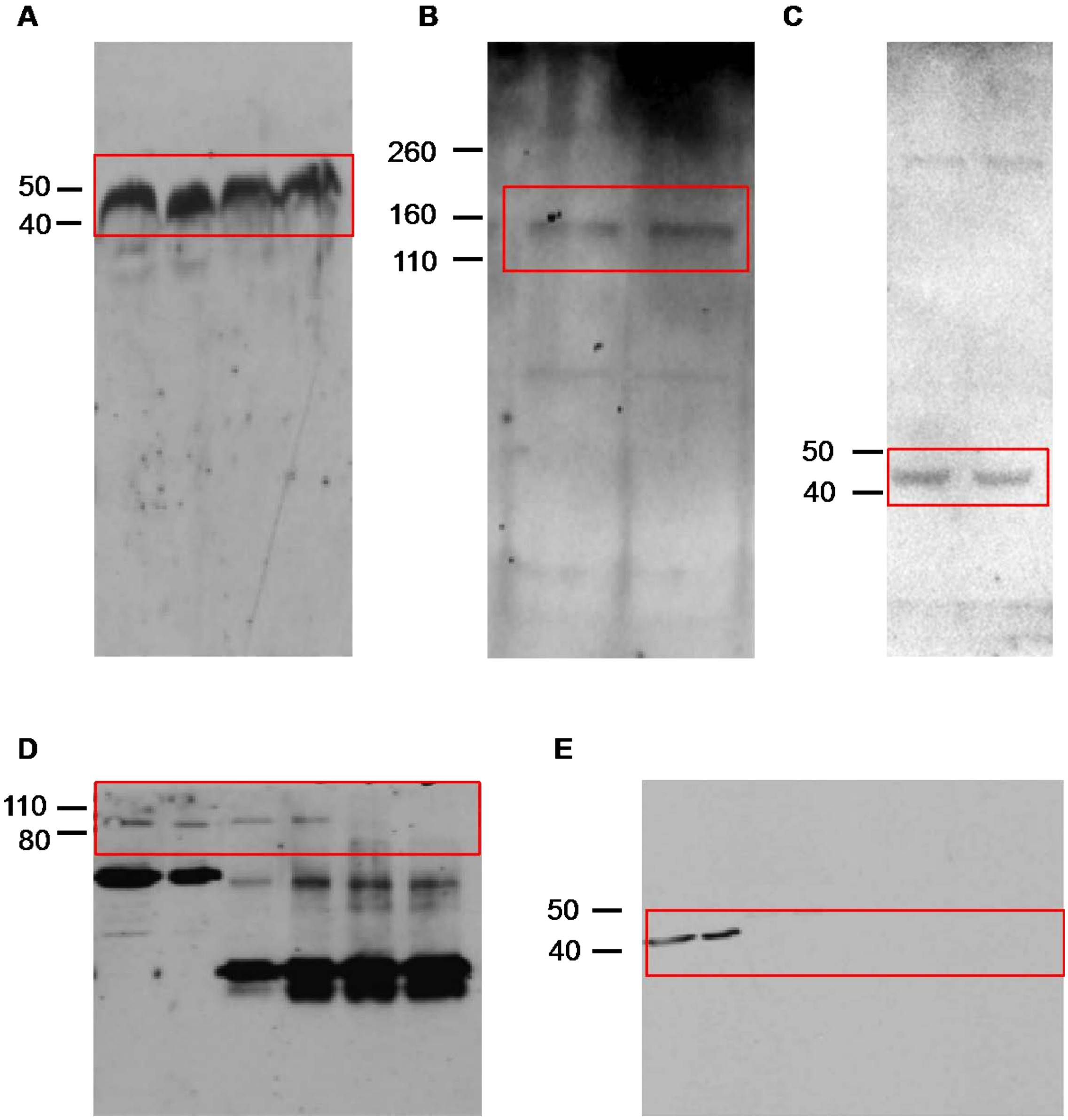

Supplemental Figure 7 – Un-cropped Western blots from main figures 1 and 2. (A) Fig. 1F CD63 **(B)** Fig. 2E SHIP1 **(C)** Fig. 2E β-Actin **(D)** Fig. 2K AGO2 **(E)** Fig. 2K β-Actin

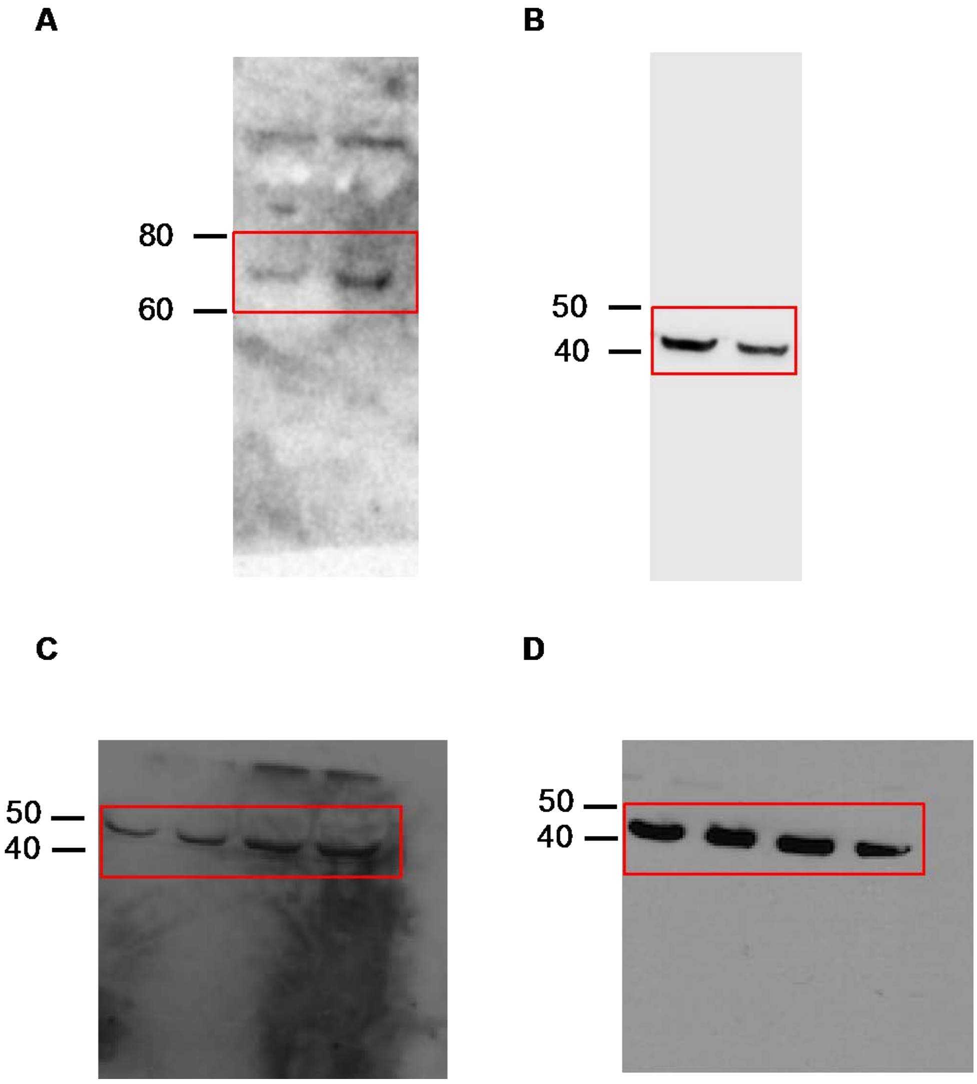

Supplemental Figure 8 – Un-cropped Western blots for main figure 3. (A) Fig. 3F IRAK1 **(B)** Fig. 3F β-Actin **(C)** Fig. 3I TRAF6 **(D)** Fig. 3I β-Actin

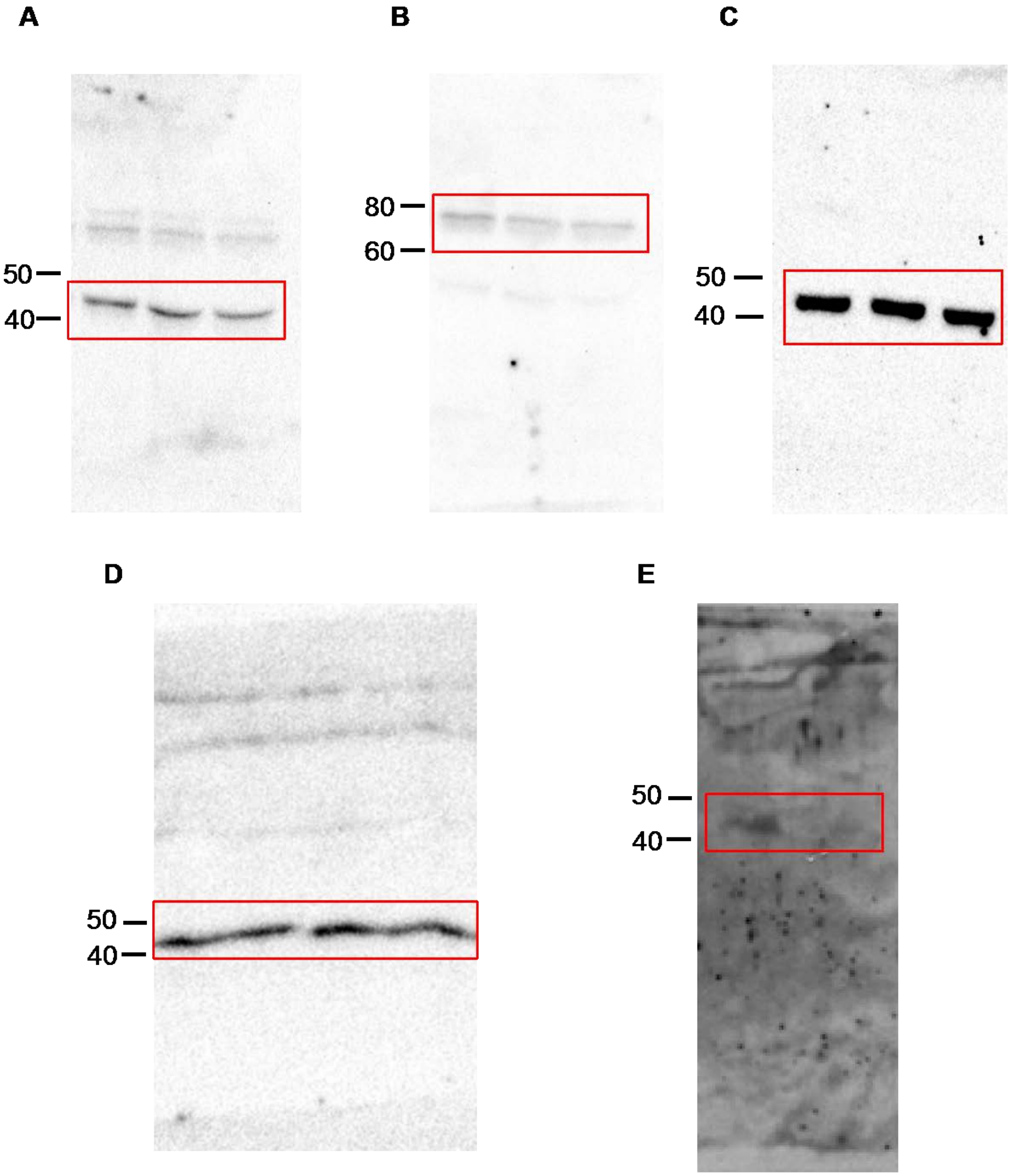

Supplemental Figure 9 – Un-cropped Western blots for main figure 4 and supplemental figure 1. (A) Fig. 4F TRAF6 **(B)** Fig. 4F IRAK1 **(C)** Fig. 4F β-Actin **(D)** Fig. 6A CD63 **(E)** Supplemental Fig. 1 CD63

A WT BMDC donor miR-155

miRbase mature miR-155

Ref	%A	%C	%G	%T	Percent coverage
U	0.02%	0.03%	0.06%	99.89%	99.22%
U	0.02%	0.02%	0.05%	99.91%	99.92%
A	99.88%	0.03%	0.06%	0.02%	99.93%
A	99.85%	0.13%	0.01%	0.01%	99.96%
U	0.06%	0.03%	0.00%	99.88%	99.96%
G	0.14%	0.07%	99.49%	0.31%	99.98%
C	0.04%	99.91%	0.02%	0.03%	100.00%
U	0.01%	0.01%	0.01%	99.97%	100.00%
A	99.92%	0.05%	0.02%	0.02%	100.00%
A	99.97%	0.01%	0.01%	0.01%	100.00%
U	0.01%	0.01%	0.00%	99.98%	100.00%
U	0.01%	0.02%	0.01%	99.97%	100.00%
G	0.03%	0.02%	99.90%	0.05%	100.00%
U	0.02%	0.03%	0.01%	99.94%	100.00%
G	0.16%	0.08%	99.65%	0.11%	100.00%
A	99.96%	0.02%	0.01%	0.02%	100.00%
U	0.01%	0.01%	0.01%	99.97%	100.00%
A	99.91%	0.04%	0.03%	0.02%	100.00%
G	0.03%	0.00%	99.95%	0.02%	99.99%
G	0.06%	0.01%	99.88%	0.05%	99.63%
G	0.25%	0.01%	99.61%	0.12%	98.52%
G	1.85%	0.24%	97.26%	0.65%	95.16%
U	3.10%	0.11%	0.66%	96.14%	88.04%
U	5.10%	0.24%	0.38%	94.28%	44.99%
U	20.08%	1.39%	1.65%	76.87%	13.29%
U	21.42%	1.66%	4.83%	72.09%	2.90%
G	20.17%	1.97%	6.34%	71.52%	0.64%

B DKO BMDC +WT exosomes miR-155

Ref	%A	%C	%G	%T	Percent coverage
U	0.33%	0.89%	0.74%	98.04%	99.09%
U	0.17%	0.25%	0.08%	99.49%	99.83%
A	99.83%	0.07%	0.10%	0.00%	99.83%
A	99.92%	0.08%	0.00%	0.00%	99.83%
U	0.62%	0.15%	0.00%	99.23%	99.83%
G	0.50%	0.15%	99.12%	0.23%	99.92%
C	0.50%	99.07%	0.18%	0.25%	100.00%
U	0.15%	0.33%	0.17%	99.34%	100.00%
A	99.61%	0.25%	0.07%	0.07%	100.00%
A	99.49%	0.44%	0.00%	0.07%	100.00%
U	0.00%	0.07%	0.15%	99.77%	100.00%
U	0.25%	0.07%	0.25%	99.42%	100.00%
G	0.40%	0.35%	98.25%	1.00%	100.00%
U	0.40%	0.25%	0.23%	99.12%	100.00%
G	0.68%	0.55%	98.04%	0.74%	100.00%
A	99.14%	0.47%	0.15%	0.23%	100.00%
U	0.00%	0.00%	0.08%	99.92%	100.00%
A	98.68%	0.88%	0.26%	0.17%	100.00%
G	0.07%	0.00%	99.93%	0.00%	100.00%
G	0.29%	0.22%	99.04%	0.45%	99.67%
G	0.27%	0.33%	98.21%	1.18%	98.51%
G	2.44%	0.39%	96.45%	0.72%	95.87%
U	3.94%	0.64%	1.37%	94.05%	89.35%
U	5.84%	0.17%	0.16%	93.83%	46.08%
U	24.67%	0.00%	2.84%	72.49%	13.63%
U	19.21%	5.29%	6.88%	68.62%	3.30%
G	12.50%	0.00%	0.00%	87.50%	1.36%

Supplemental Table 1 – miR-155 nucleotide differences between Wt donor BMDCs and DKO BMDCs treated with Wt exosomes. (A) Mature miR-155 nucleotide composition was analyzed in Wt BMDCs using RNA-Seq. Red represents significant changes between Wt donors and DKO BMDCs that received Wt exosomes (n=3). **(B)** Mature miR-155 nucleotide composition in DKO BMDCs that received Wt exosomes as determined by RNA-Seq. Red represents significant changes between Wt donors and DKO BMDCs that received Wt exosomes (n=3).

A WT BMDC donor miR-146a

mIRbase mature miR-146a

Ref	%A	%C	%G	%T	Percent coverage
U	0.01%	0.02%	33.37%	66.60%	99.96%
G	0.07%	0.02%	99.87%	0.04%	100.00%
A	66.62%	0.02%	0.03%	33.33%	100.00%
G	33.34%	0.02%	66.62%	0.02%	100.00%
A	66.60%	33.26%	0.05%	0.07%	100.00%
A	66.53%	33.35%	0.06%	0.07%	100.00%
C	0.02%	66.63%	0.02%	33.33%	100.00%
U	0.01%	0.01%	0.01%	99.98%	100.00%
G	33.34%	0.01%	66.62%	0.03%	100.00%
A	99.97%	0.01%	0.01%	0.01%	100.00%
A	66.66%	0.01%	33.33%	0.01%	100.00%
U	0.01%	0.01%	0.00%	99.97%	100.00%
U	0.01%	33.33%	0.01%	66.66%	100.00%
C	33.26%	66.64%	0.05%	0.05%	100.00%
C	33.36%	66.44%	0.09%	0.11%	100.00%
A	66.66%	0.01%	33.31%	0.02%	100.00%
U	33.31%	0.02%	0.02%	66.65%	99.99%
G	0.13%	0.02%	99.80%	0.06%	99.99%
G	0.01%	0.01%	99.93%	0.04%	99.98%
G	0.03%	0.04%	66.60%	33.33%	99.94%
U	0.32%	0.14%	0.04%	99.49%	99.69%
U	2.02%	0.20%	0.39%	97.39%	94.42%
A	40.89%	1.25%	2.05%	55.81%	41.04%
U	39.65%	0.77%	3.71%	55.87%	11.63%
A	63.63%	1.84%	3.21%	31.32%	1.39%
U	15.77%	0.98%	4.90%	78.35%	0.22%

B DKO BMDCs + WT exosomes miR-146a

Ref	%A	%C	%G	%T	Percent coverage
U	0.00%	0.87%	0.43%	98.70%	100.00%
G	0.00%	0.43%	99.25%	0.32%	100.00%
A	100.00%	0.00%	0.00%	0.00%	100.00%
G	0.00%	0.00%	100.00%	0.00%	100.00%
A	100.00%	0.00%	0.00%	0.00%	100.00%
A	99.57%	0.43%	0.00%	0.00%	100.00%
C	0.00%	99.68%	0.32%	0.00%	100.00%
U	0.00%	0.74%	0.00%	99.26%	100.00%
G	0.00%	0.00%	100.00%	0.00%	100.00%
A	99.68%	0.00%	0.32%	0.00%	100.00%
A	100.00%	0.00%	0.00%	0.00%	100.00%
U	0.74%	0.00%	0.00%	99.26%	100.00%
U	0.00%	0.00%	0.00%	100.00%	100.00%
C	0.00%	100.00%	0.00%	0.00%	100.00%
C	0.00%	99.68%	0.00%	0.32%	100.00%
A	99.25%	0.32%	0.43%	0.00%	100.00%
U	0.00%	0.74%	0.00%	99.26%	100.00%
G	21.65%	4.27%	73.44%	0.64%	100.00%
G	0.00%	0.00%	100.00%	0.00%	100.00%
G	0.32%	1.07%	97.87%	0.74%	100.00%
U	1.73%	21.64%	0.00%	76.63%	99.56%
U	3.73%	0.70%	0.35%	95.22%	91.15%
A	60.41%	0.51%	8.50%	30.58%	58.85%
U	15.67%	0.00%	2.75%	81.58%	32.30%
A	95.45%	0.00%	0.00%	4.55%	19.03%
U	5.56%	0.00%	0.00%	94.44%	11.06%

Supplemental Table 2 – miR-146a nucleotide differences between Wt donor BMDCs and DKO BMDCs treated with Wt exosomes. (A) Mature miR-146a nucleotide composition in Wt BMDCs donors as determined by RNA-Seq. Red represents significant changes between Wt donors and DKO BMDCs that received Wt exosomes (n=3). **(B)** Mature miR-146a nucleotide composition in DKO BMDCs that received Wt exosomes as determined by RNA-Seq. Red represents significant changes between Wt donors and DKO BMDCs that received Wt exosomes (n=3).
